# A systematic review of in vivo brain insulin resistance biomarkers in humans

**DOI:** 10.1016/j.bionps.2025.100125

**Published:** 2025-06

**Authors:** Graham Reid, Brendan Sargent, Sarah Bauermeister, Amanda Adler, Ivan Koychev

**Affiliations:** a Department of Psychiatry, Medical Sciences Division, University of Oxford, Oxford, United Kingdom; b Radcliffe Department of Medicine, Medical Sciences Division, University of Oxford, Oxford, United Kingdom

**Keywords:** Brain insulin resistance, Dementia, Extracellular vesicles, Neuroimaging

## Abstract

Type 2 diabetes mellitus (T2DM) is associated with an elevated risk of dementia, prompting interest into the concept of brain-specific insulin resistance. However, the brain's reliance on insulin-independent glucose transporters complicates attempts to measure in vivo brain insulin resistance using the definition of system-wide insulin resistance, which is based on glucose-insulin interactions. In this review, we explore three available biomarkers for evaluating in vivo brain-specific insulin resistance in humans: (1) correlating systemic insulin resistance with brain function, (2) examining functional brain changes after the administration of intranasal insulin, and (3) quantifying insulin signalling proteins in neuronally enriched blood-derived extracellular vesicles. Integrating evidence from these three approaches tentatively suggests for the first time that a comprehensive assessment of the brain's default mode network (DMN), combining these methodologies within a single study, may offer a useful biomarker to quantify in vivo brain-specific insulin resistance in humans. Correlating DMN responses to concentrations of pY-IRS-1 in blood-derived extracellular vesicles would corroborate evidence for a brain-specific biomarker and provide a scalable approach to detecting brain-specific insulin resistance in humans. This advancement would enable in vivo evaluations of insulin resistance in the central nervous system, akin to the precise measurements of systemic insulin resistance seen in T2DM. An established and clearly defined biomarker of in vivo brain insulin resistance in humans would permit further investigation into the links between diabetes and dementia, ultimately bolstering support for secondary dementia prevention by identifying those at higher risk for cognitive decline.

## Highlights

• A single, validated, and universally agreed biomarker of in vivo brain-specific insulin resistance in humans is lacking.
• We review evidence of three possible methods for measuring insulin resistance in the human brain.
• The default mode network (DMN) may be a key candidate biomarker for measuring brain-specific insulin resistance.
• pY-IRS-1 in neuronally enriched extracellular vesicles may be a scalable biomarker of brain-specific insulin resistance.
• pY-IRS-1 should be correlated with the DMN's association with peripheral insulin resistance and intranasal insulin response.

## Introduction

Dementia is a syndrome of cognitive dysfunction that affects over 50 million people worldwide (Shin, 2022). There is currently no cure for dementia, but research has shown that some risk factors can be modified to help mitigate the likelihood of developing the condition (Livingston et al., 2020). One such risk factor is type 2 diabetes mellitus (T2DM), which is characterised by insulin resistance (IR) in which the body's cells do not respond normally to insulin (Albai et al., 2019, Li et al., 2022). This results in higher levels of glucose in the blood, leading to further secretion of insulin from the pancreas, which is associated with brain damage and may explain T2DM’s contribution of 1–3 % of worldwide dementia cases (Bahniwal et al., 2017, Livingston et al., 2020, Yang et al., 2022). Given the relationship between the action of glucose and insulin, IR can be assessed in vivo by measuring the relative levels of glucose and insulin in the blood (Gutch et al., 2015). A high level of glucose relative to insulin is the agreed biomarker of system-wide IR.

Although the brain was previously thought to be an insulin-insensitive organ, recent research has suggested that this is not the case (Milstein & Ferris, 2021). There is growing evidence that the brain is sensitive to insulin, and that brain-specific IR may be a risk factor for dementia (Kellar & Craft, 2020). One challenge in studying brain IR, however, is that the system-wide (whole body) definition of IR, which is based on elevated levels of glucose, does not easily apply to the central nervous system. This is because most glucose uptake in the brain is mediated by insulin-independent glucose transporters (McEwen & Reagan, 2004). In addition, it is also known that it is possible to be brain IR independent of system-wide IR, indicating that brain and body IR are not necessarily linked (Talbot et al., 2012). As a result, defining and measuring brain-specific IR requires a biomarker, which is not necessarily tied to whole body glucose metabolism. Such a biomarker of brain-specific IR would allow researchers to further explore the relationship between IR and dementia (Sarah et al., 2023). This would help to identify the extent to which system-wide IR and brain IR are separable, and to identify people with T2DM who may be at higher risk for dementia if they have comorbid brain-specific IR. This would help to improve our understanding of the mechanisms underlying dementia and its relationship to T2DM, and to develop new strategies to prevent and treat dementia. In a recent review paper, it was shown that in vivo imaging and blood biomarkers (in addition to cognitive responses to insulin) are some of the available biomarkers to probe brain IR (Elizabeth M. Rhea et al., 2023). However, to date a review of the neuroimaging responses to intranasal insulin, the neuroimaging correlates of body-wide IR, and blood biomarkers has not been conducted.

In this systematic review, we discuss three existing biomarkers that could be used to define in vivo IR in human brains. We highlight that although these strategies collectively point to the existence of brain IR, there is no single validated measurement of in vivo IR for the central nervous system in humans, as is the case with the system-wide IR seen in T2DM. By synthesising results across these three methods, we tentatively suggest that a detailed investigation of the default mode network, using these three methods in a single design, may lead to a standardised and validated biomarker of in vivo brain IR in humans. With these findings, we present suggestions for the field in terms of how a brain-specific definition of IR may be established and how an assessment of brain IR may be scaled up by using blood biomarkers to help identify people at risk for dementia in a cost-effective way.

## Brain-specific insulin resistance

### Evidence of insulin action in the brain

Recent research has challenged the previous belief that the brain is insensitive to insulin. It has been discovered that hippocampal and olfactory bulb progenitor cells are capable of producing and releasing insulin de novo (Kuwabara et al., 2011). In a study by Mazucanti et al. (2019), it was shown that insulin is also secreted by the choroid plexus, which is a brain structure near the ventricles that produces most of the nervous system’s cerebrospinal fluid. Moreover, there is evidence of cortical production of insulin in GABAergic neurogliaform cells (Molnár et al., 2014). Insulin mRNAs have been detected in endothelial and glial cells in human and mouses brains (He et al., 2018, Vanlandewijck et al., 2018, Yang et al., 2022, Zhang et al., 2020), as well as in the periventricular nucleus of the hypothalamus and in neonatal rabbit brains, such as in the hippocampus (i.e., CA1, CA3, and the dentate gyrus) (Devaskar et al., 1994, Young, 1986). In human brains, C-peptide, which is associated with local insulin production, has been found in the hypothalamus in higher concentrations than in the blood, suggesting extra pancreatic insulin production (Dorn et al., 1983). In post-mortem human brain tissue, insulin mRNA transcripts have been found in the hippocampus and hypothalamus, which also provides evidence of possible insulin synthesis in the human central nervous system (Steen et al., 2005). That said, insulin levels in cerebrospinal fluid are much lower than insulin levels in plasma, but they are correlated, which suggests that most insulin in the central nervous system is derived from pancreatic insulin in the blood (Wallum et al., 1987). Relatedly, insulin has been found to traverse the blood-brain barrier through a specialised carrier located on the vascular endothelium, specifically the capillary endothelial cells (Pemberton et al., 2022, Wang et al., 2022). This implies that insulin receptors are present in various regions of the brain and studies have found high density expression of insulin receptors in the hypothalamus, hippocampus, cerebellum, striatum, olfactory bulb, and neocortex (Adamo et al., 1989, Marks et al., 1990, Unger and Betz, 1998, Unger et al., 1989, Werther et al., 1987). More recently, insulin has been shown to be involved in synaptic viability and regeneration following axonal damage. For example, studies have shown that insulin promotes the repair of postsynaptic sites and reconnection with presynaptic targets, resulting in reduced neuronal death (Agostinone et al., 2018). Insulin has also been shown to mediate pre- and postsynaptic neuroplasticity in hippocampal glutamatergic synapses (Shypshyna et al., 2023). There is also growing evidence that insulin signalling supports cell proliferation and neurogenesis in zebrafish following intracerebroventricular injection (Gence et al., 2023). Similarly, in animal models of neuroinflammation, there is evidence that insulin has an anti-inflammatory effect in the hippocampus, which is associated with improved visuospatial learning and memory (Adzovic et al., 2015).

### Evidence of insulin resistance in dementia

In the context of dementia, post-mortem studies have revealed that the brains of individuals with Alzheimer's disease (AD) exhibit sings of IR. For example, in post-mortem brain tissue of patients with AD, studies have found inhibited activation of the insulin receptor and insulin-stimulated phosphorylation of the molecules involved in the insulin signalling pathways, which is not seen in healthy age- and sex-matched controls (Talbot et al., 2012). In patients with AD or mild cognitive impairment (MCI), phosphorylation of the insulin receptor substrate is higher than in healthy controls and these abnormalities have been found to associate with the AD biomarkers, as well as scores of cognitive ability (Arvanitakis et al., 2020, O'Neill et al., 2012, Tramutola et al., 2015). In animal models of tauopathy, studies have shown that brain IR mediates changes in the hyperphosphorylation of tau via changes to downstream crosstalk in the insulin receptor pathway (Chatterjee et al., 2019). In light of these findings, researchers have put forth the idea that targeting brain-specific IR in individuals at risk for AD could potentially yield preventive, or disease-modifying, effects. There is some, inconclusive, evidence that the intranasal administration of insulin directly to the brain may support cognitive abilities, such as verbal episodic recall (Long et al., 2022). Longitudinally, intranasal insulin for 16 weeks seems to support cognition and to sustain functional abilities in people with MCI and AD in which cognitive changes seem to correlate with cerebrospinal fluid biomarkers of AD (Craft et al., 2012). Together, these findings suggest the possibility of a key role for brain IR in the pathogenesis of AD or conversion of AD to dementia.

### Challenges in measuring brain-specific insulin resistance

The challenge for the field is that a validated and reliable biomarker of in vivo IR in the human central nervous system does not exist. This is in direct contrast to the literature on T2DM where several established measures of systemic insulin sensitivity are available, including the gold-standard hyperinsulinemic-euglycemic clamp technique (HEC), the homeostatic model assessment of IR (HOMA-IR), and various forms of glucose tolerance tests (GTT)(Gastaldelli, 2022). Underpinning the validity of these tools in measuring insulin sensitivity in the body’s peripheral organs is the observation that glucose levels in IR remain elevated irrespective of increasing levels of insulin. Thus, built into the definition of systemic IR is a deviation in the body’s typical response to insulin whereby glucose levels normally drop in response to elevated insulin circulation (i.e., IR is defined as a reduction in the insulin-mediated uptake of glucose). In the brain, however, glial and neuronal uptake of glucose is mediated primarily by the insulin-independent glucose transporters, GLUT-1 and GLUT-3, respectively (Mantych et al., 1992, Morgello et al., 1995). This means that a biomarker of brain IR that goes beyond or nuances the glucose-based definition of system-wide IR may be useful. Furthermore, the insulin receptor gene differentially splices into long and short isoforms of the receptor, which are distributed differently throughout the body. The long isoform, known as insulin receptor B (IR-B), preponderates in the body’s adipose, renal, muscular, and hepatic tissues, playing a role in insulin’s metabolic effects in the periphery (Beneit et al., 2016). Within the central nervous system, IR-B, along with the short isoform, known as insulin receptor A (IR-A), are expressed across different cell types. However, while glial cells, such as astrocytes, express both isoforms, neurons only express the short isoform of the insulin receptor (in contrast to the expression of the long isoform in the periphery that is involved in metabolism)(Belfiore et al., 2017). As such, there is precedence to look at the possibility of distinct definitions, and by extension biomarkers, of IR in the body and IR in the brain.

### Currently available biomarkers of in vivo brain-specific insulin resistance in humans

Here we show that researchers currently have three possible means of measuring in vivo brain-specific IR in humans. The first involves correlating system-wide IR with brain function using neuroimaging techniques. The second approach involves delivering insulin to the central nervous system with the use of a nasal spray while assessing changes in brain activity. And the third approach in measuring brain-specific IR in humans has been to measure proteins involved in the insulin signalling pathway in neuronally-enriched blood-derived extracellular vesicles.

#### Aim of the current study

To date, there has been no review synthesising possible evidence of in vivo brain-specific IR in humans across these three biomarkers. As such, the purpose of this review was to review studies across these three methods together for the first time. It is hoped that such a synthesis will capture the ways in which brain-specific IR has been measured, perhaps pointing to a clearly defined biomarker of brain-specific IR, as is seen in system wide IR.

## Methods

The protocol for this systematic review was registered on PROSPERO (International Prospective Register of Systematic Reviews) with the ID number CRD42022369807. In writing this manuscript, PRISMA reporting guidelines have been followed throughout.

### Data sources and search strategy

A systematic literature search was performed on MEDLINE (Ovid), EMBASE (Ovid), PsycINFO (Ovid), CINAHL (EBSCOhost), ProQuest Dissertations and Theses Global (ProQuest), the Cochrane Database of Systematic Reviews, and the Cochrane Central Register of Controlled Trials. Each database was searched for human studies from inception to the 7th of December 2022 without any restrictions placed on language. In order to identify relevant ongoing and completed trials, we also searched several clinical trial registries, including ClinicalTrials.gov, Current Controlled Trials, Australian New Zealand Clinical Trials Registry, University Hospital Medical Information Network Clinical Trials, and the International Clinical Trials Registry Platform. And lastly, we manually screened the reference lists of included studies to identify additional papers for inclusion in the review. Following PICO guidelines (Population, Intervention, Comparison, and Outcome specifications), we were interested in neuroimaging measures of sensitivity to insulin in both healthy and patient populations. No restrictions were placed on the measurement of insulin sensitivity, the neuroimaging modality used to define IR in the central nervous system, nor the insulin-signalling proteins measured in extracellular vesicles that had been enriched for neuronal origin. A senior information specialist at the University of Oxford peer reviewed our electronic search strategy (PRESS) prior to execution. Details of the search strategy are included in supplementary material 1.

### Inclusion and exclusion criteria

Included publication types were cross-sectional and longitudinal randomised control trials, experimental medicine studies, and correlational observation studies. Excluded publication types included studies that did not investigate a functional neuroimaging response or correlate of insulin sensitivity, studies that did not investigate insulin sensitivity or resistance, studies that were not conducted with humans, studies that were written in any language other than English, qualitative studies, case reports, papers written to review existing literature, abstracts submitted to academic conferences, papers that had not been peer-reviewed in academic journals, and manuscripts submitted to journals as opinion pieces or editorials.

### Data selection and synthesis

#### Study selection

To identify potentially eligible studies, two independent reviewers (GR and BS) ran our search strategy on the above databases. Records were initially deduplicated using EndNote’s duplicate identification function. Where identical records from different databases produced distinct entries in EndNote, deduplication was performed manually. Based on our inclusion criteria, both reviewers independently screened the title and abstract of the records in Rayyan. Any disagreements on the final selection of records for full-text screening were resolved through discussion with a third reviewer (IK).

#### Data extraction

A single reviewer (GR) extracted details about the records’ author and title, population group, participant demographics, measurement of insulin sensitivity, neuroimaging marker of brain IR, insulin-signalling protein, study design, and main findings (see supplementary material 2). The extracted data were reviewed by a second researcher (BS) and any disagreements were resolved through discussion with a third reviewer (IK).

#### Data synthesis

The findings were synthesised narratively, grouping studies around the measurement of insulin sensitivity and functional neuroimaging techniques used to suggest in vivo brain-specific IR in humans. This resulted in a narrative section for brain correlates of peripheral IR, one for the brain’s responsiveness to intranasal insulin, and a separate section for studies reporting insulin signalling in neuronally-enriched extracellular vesicles.

### Risk of bias

NIH Quality Assessment Tools were applied by two reviewers (GR and BS) to assess the risk of bias in the eligible records. Where any disagreement was found, a third reviewer (IK) was consulted. Across the tools’ 14 questions, including whether the research question, design, sample, and materials were clearly justified, each paper was assigned a poor, fair or good quality rating.

## Results

The PRISMA diagram showing the process of screening and selecting eligible studies is shown in supplementary material 3. Of the 6949 studies that were identified in the initial search, 254 met the eligibility criteria during title-abstract screening. Following a full-text review of 212 papers, a total of 82 records were retained for inclusion in this systematic review. The NIH Quality Assessment Tool revealed that no studies had a poor-quality rating, 38 studies had a fair quality rating, and 44 papers had a good quality rating (see supplementary material 4). No papers were excluded from this review based on the risk of bias. Of the final sample of 82 papers, 47 correlated peripheral insulin sensitivity with functional brain outcomes (supplementary material 5). For tables showing emerging patterns of results in brain regions or networks with growing evidence of an association with peripheral IR, see Table 1 and Table 2. 25 studies administered intranasal insulin to the central nervous system directly while assessing the neuroimaging responses (supplementary material 6). For tables showing emerging patterns of results in brain regions or networks with growing evidence of responsiveness to intranasal insulin administration, see Table 3 and Table 4. A total of 10 papers quantified molecules involved in the insulin signalling pathway in neuronally-enriched extracellular vesicles (supplementary material 7). Of the 47 papers measuring peripheral IR, 1 used a quantitative insulin-sensitivity check index (QUICKI), 32 used HOMA-IR (in which one also measured QUICKI), 9 used the HEC technique, and 5 used a glucose tolerance test. Of the neuroimaging techniques used across the 72 papers, 21 used positron emission tomography (PET), 18 papers used resting-state fMRI (rs-fMRI), 15 used tasked-based fMRI, 10 used arterial spin labelling (ASL), 2 used electroencephalography (EEG), 4 used magnetoencephalography (MEG), 1 used doppler ultrasound, and 1 used single-photon emission computed tomography (SPECT).

**Table 1:** Number of significant associations reported between peripheral insulin resistance and commonly reported brain areas.

**Brain Area**	***N*****of Significant Findings Reported**
Frontal Lobe	35
Parietal Lobe	31
Basal Ganglia	12
Temporal Lobe	12
Insula	4
Thalamus	3
Midbrain	2
Occipital Lobe	2
Amygdala	1
Diencephalon	1
Cerebrum	1

**Table 2:** Table of key takeaways for studies associating peripheral insulin resistance with measures of brain activity.

**Strength of Evidence**	**Brain Focus**	**General Pattern of Findings**	**Notes**
**Strong Evidence**	**Default Mode Network** *Posterior cingulate cortex, precuneus, hippocampus*	↓ Connectivity, ↓ glucose metabolism, ↑ activity viewing foods, ↓blood flow, ↓ resting state activity, ↓ activity during working memory tasks	Multiple studies show associations in these regions
	**Basal Ganglia** *Striatum, caudate, putamen, nucleus accumbens*	↓ Dopamine availability, ↑ connectivity, ↓ glucose metabolism	Multiple studies show associations in these regions
**Moderate Evidence**	**Salience and Reward Networks** *Anterior cingulate cortex, insula, orbitofrontal cortex*	↑ Connectivity in reward-related regions, ↑ activity responses to reward cues	Mixed results but trends towards positive associations in responds to rewards
	**Frontotemporal Regions**	↓ Glucose metabolism, ↓ blood flow, ↓ connectivity	Results trending towards negative associations across imaging modalities
**Weak Evidence**	**Electroencephalography**	Unclear pattern of results	Low number of studies with inconsistent findings

**Table 3:** Number of significant effects reported of intranasal insulin administration across commonly reported brain areas.

Brain Area	*N* of Significant Findings Reported
Frontal Lobe	8
Basal ganglia	8
Temporal Lobe	6
Hippocampus	6
Parietal Lobe	5
Insula	4
Hypothalamus	4
Amygdala	3
Occipital Lobe	2
Ventral Tegmental Area	2
Thalamus	1

**Table 4:** Table of key takeaways for studies looking at the effect of intranasal insulin on brain responses.

**Strength of Evidence**	**Brain Focus**	**General Pattern of Findings**	**Notes**
**Strong Evidence**	**Default Mode Network** *Posterior cingulate cortex, precuneus, hippocampus, medial prefrontal cortex*	↑ connectivity, ↑ blood flow, ↓ cerebrovascular reactivity	Multiple studies show effects in these regions
**Moderate Evidence**	**Salience and Reward Networks** *Anterior cingulate cortex, insula, orbitofrontal cortex*	↑ food cue responses, ↑ connectivity, ↑ blood flow	Results tending towards effects in these areas
**Weak Evidence**	**Basal Ganglia** *Striatum, caudate, putamen, nucleus accumbens*	∼ activity, ∼ blood flow, ↓ dopamine availability	Multiple studies reporting effects in these regions, but directionality tends to be mixed

*Note*. Given the large number of findings across brain areas, networks, and regions, results were grouped according to higher-order category where possible to give an indication of the most commonly reported areas showing a significant association with peripheral IR.

### Brain correlates of peripheral measures of insulin resistance

#### Electro- and magnetoencephalography for brain activity

Two studies investigated the brain correlates of peripheral IR with EEG. In one study, there was a positive association between peripheral IR and frontal asymmetry towards the right hemisphere (Wolf et al., 2018). In another study, there was no evidence of any EEG signatures that associated with peripheral IR (Akin et al., 2017). One study measured the brain correlates of peripheral IR with MEG. There was evidence that higher IR was associated with lower theta activity, but there was no association with beta activity (Tschritter et al., 2006).

#### Positron emission- and single photon emission tomography for neurotransmitter behaviour

Two studies looked at the brain correlates of peripheral IR with tomography techniques used to capture serotonin and dopamine activity. In one study, higher peripheral IR was associated with lower availability of type 2 dopaminergic neurons in the left ventral striatum (Julia P. Dunn et al., 2012). In another study, there was no evidence of an association between peripheral IR and dopaminergic transporter binding (Versteeg et al., 2017). As for serotonin, higher peripheral IR was associated with lower serotonin transporter binding in the diencephalon, but not the hypothalamus (Versteeg et al., 2017).

#### Resting-state functional brain activity for brain network activity

**Reward and saliency networks:** In one study, peripheral IR, after eating but not when fasted, was associated with greater connectivity between the caudate and the putamen and lower network connectivity with the dorsal anterior cingulate cortex (Ryan et al., 2018). In another study, higher system-wide IR was associated with higher connectivity in the putamen and orbitofrontal cortex (Kullmann et al., 2012). And in a third study, higher peripheral IR was associated with higher connectivity between the ventral striatum and anterior insula(Ryan et al., 2012).

**Default mode network:** Across three studies, higher IR was associated with lower connectivity in regions of the default mode network, including the posterior cingulate cortex (Cui et al., 2015), as well as connectivity between the posterior cingulate cortex and (i) the right middle temporal gyrus, the left lingual gyrus, the left middle occipital gyrus, and the left precentral gyrus (Chen et al., 2014) and (ii) the right inferior frontal gyrus and right precuneus (Musen et al., 2012). As for the precuneus, another region of the DMN, one study found that higher IR was associated with lower activity in this region (Mazza et al., 2022), while another study reported a null finding (Cui et al., 2015). And lastly, in one study, higher IR was associated with lower short-range connectivity in the left medial orbitofrontal cortex (Lopez-Vilaret et al., 2022), while no associations were reported with connectivity in the bilateral superior frontal gyrus(Cui et al., 2015).

**Networks in diabetic complications:** Two studies investigated the relationship between resting-state brain activity and IR in T2DM with retinopathy. One study found a positive association between IR and the degree distribution (an inverse measure of network node connectivity) in the right middle occipital gyrus, but a negative association with the right superior orbital frontal gyrus, right middle frontal gyrus, right rolandic operculum, right insula, and bilateral angular gyrus (Dai et al., 2017). In contrast, another study found no association between IR and activity in the bilateral occipital gyri, right lingual gyrus, precuneus, right posterior/anterior cerebellar lobe, or parahippocampal, fusiform, superior temporal, inferior parietal, or angular gyrus (Wang et al., 2017). One study investigated the relationship between resting state brain activity and IR in T2DM with neuropathy, finding a negative association between IR and resting state activity in (i) the left middle occipital lobe in patients with painful diabetic neuropathy and (ii) the left postcentral gyrus in T2DM without painful neuropathy (Zhang et al., 2019).

**Interhemispheric connectivity:** One study investigated the relationship between IR and interhemispheric connectivity as a brain correlate of IR, finding that higher IR was associated with reduced connectivity between the bilateral middle temporal gyri (Xia et al., 2015).

#### Task-based functional brain activity for regional brain activity during cognitive tasks

**Working memory:** Across two studies, people with higher peripheral IR had lower activity in the parietal cortex during working memory tasks, including the right parietal cortex (Gonzales et al., 2010) and bilateral precuneus (Williams et al., 2019).

**Episodic memory:** In one study, higher peripheral IR was associated with higher activity in the frontal, precentral, and inferior frontal gyrus during recognition tasks of non-food items (Marder et al., 2014). However, in another type of episodic memory test, known as an olfactory recognition test, IR was not related to whole-brain activity or activity in the putamen, caudate, nucleus accumbens or orbitofrontal cortex (Poessel et al., 2022).

**Executive function:** In one study, higher peripheral IR was positively associated with activity in the right precuneus, right thalamus, left supplementary motor area, and right precentral gyrus, but negatively associated with activity in the right putamen during a test of impaired inhibition (Eckstrand et al., 2017).

**Visual perception:** Several studies showed a positive relationship between peripheral IR and activity in brain regions when looking at food cues, including the left nucleus accumbens and right cingulate gyrus (Drummen et al., 2019), left mid cingulate gyrus (Wever et al., 2021), right anterior cingulate cortex, left ventral tegmental area, bilateral medial prefrontal cortex, bilateral midbrain, and the right caudate (Alsaadi & Van Vugt, 2015). Activity in other brain regions of the forebrain including the right rolandic operculum (Wever et al., 2021) and bilateral insula (Drummen et al., 2019) were also found to positively associate with peripheral IR, while activity in the dorsolateral prefrontal cortex seems to have an inverse relationship with system-wide IR (Alsaadi & Van Vugt, 2015). When viewing high calorie foods, one study reported a negative association between peripheral IR and activity in the putamen, but a positive association between IR and brain activity in the inferior parietal lobule, posterior cingulate cortex, anterior cingulate cortex, precentral gyrus, middle frontal gyrus, middle temporal gyrus, superior frontal gyrus, precuneus, medial frontal gyrus and left cerebrum (Van Vugt et al., 2014). When viewing low calorie foods, this study found that participants showed a positive association between IR and activity in the precuneus, posterior cingulate cortex, superior frontal gyrus, superior temporal gyrus, and rolandic operculum (Van Vugt et al., 2014).

#### Measures of cerebral blood flow

In one study higher IR was related to lower cerebrovascular reactivity across the bilateral middle cerebral arteries (Rodriguez-Flores et al., 2014). Cerebrovascular reactivity is a measurement of the brain’s ability to adapt blood supply to meet its metabolic demands (Wang et al., 2023). In the frontal lobe, higher peripheral IR was associated with lower CBF across two studies in regions, such as the left middle frontal gyrus, right anterior cingulate, right precentral gyrus, right medial frontal gyrus (Hoscheidt et al., 2017), right orbitofrontal cortex, right superior frontal gyrus, and the left postcentral gyrus (Thambisetty et al., 2013). In one study, the temporal lobe, higher peripheral IR was associated with lower CBF in the right temporal lobe (Hoscheidt et al., 2017) and the left superior temporal gyrus (Thambisetty et al., 2013). In the parietal lobe, higher peripheral IR was associated with lower CBF in the posterior cingulate cortex, precuneus (Cui et al., 2017), and right inferior parietal lobule (Thambisetty et al., 2013). Positive associations were also reported, however, in the left inferior frontal and left precentral gyrus, the left middle temporal gyrus, and left globus pallidus of the basal ganglia (Thambisetty et al., 2013).

#### Brain glucose metabolism on FDG positron emission tomography

**Whole brain:** Across three studies, higher IR, when measured using HOMA-IR, was associated with lower whole-brain glucose metabolism (Anthony et al., 2006, Chen et al., 2022, Willette et al., 2015). When using the gold-standard HEC technique, however, 6 studies found that higher IR, as measured by lower glucose infusion rates, was associated with higher whole-brain glucose uptake (Boersma et al., 2018, Eriksson et al., 2021, Honkala et al., 2018, Johansson et al., 2018, Latva-Rasku et al., 2018, Rebelos et al., 2021). And in one study, there was no difference in peripheral IR between people with low and normal levels of brain glucose uptake (Nam et al., 2017).

**Frontal lobe:** Four studies reported associations between peripheral IR and glucose metabolism in the frontal lobe. For example, higher IR was associated with lower glucose metabolism in the prefrontal cortex (Anthony et al., 2006), bilateral frontal lobes (Baker et al., 2011), superior frontal cortex (Castellano et al., 2015), bilateral middle frontal cortex (Chen et al., 2022) (for cf. see (Castellano et al., 2015)), right inferior frontal gyrus and the right precentral gyrus (Chen et al., 2022). However, in one study, higher IR was associated with higher, and not lower, glucose metabolism in the inferior prefrontal gyrus and premotor cortex (Boersma et al., 2018) with no association between IR and the most anterior part of the prefrontal cortex (i.e., the frontal pole)(Castellano et al., 2015).

**Temporal lobe:** In the temporal lobe, higher peripheral IR was associated with lower glucose metabolism in the left temporal lobe (Baker et al., 2011) and bilateral middle temporal lobes (Castellano et al., 2015) (for cf. see (Willette et al., 2015)), including regions such as the left (Chen et al., 2022, Garcia-Casares et al., 2014) and right middle temporal gyri (Chen et al., 2022). In contrast to the hypometabolism of the left middle temporal lobe reported elsewhere (Castellano et al., 2015), one study found that higher IR was associated with increased glucose metabolism in the same region (Willette et al., 2015). In superior and inferior temporal regions, Castellano et al., (2015) reported no association with IR. The researchers also found no relationship between IR and the entorhinal cortex or parahippocampal gyrus. In a study by Garcia-Casares et al., (2014), higher IR was associated with lower glucose metabolism in the left insula, which separates the temporal lobe from the frontal and parietal lobes. In another study, higher IR was associated with higher glucose metabolism in the fusiform gyrus (Boersma et al., 2018).

**Parietal lobe:** Two studies found a negative association with system-wide IR and parietal brain regions. These brain areas included the left (Castellano et al., 2015) and right parietal lobes (Baker et al., 2011, Castellano et al., 2015), posterior and medial cingulate cortex (Baker et al., 2011) (cf.(Castellano et al., 2015)), and the precuneus (Baker et al., 2011) (cf.(Ishibashi et al., 2017)). Unlike the negative associations outlined above, higher IR has also been associated with higher glucose metabolism in the somatosensory association cortex and angular gyrus (Boersma et al., 2018). That said, null findings have also been reported in the parietal lobe in which glucose metabolism in the temporoparietal region (Ishibashi et al., 2017) and supramarginal gyrus (Castellano et al., 2015) showed no relationship with peripheral IR.

**Subcortical structures:** Five studies investigated the relationship between system-wide IR and glucose metabolism in subcortical structures. For example, higher IR was associated with lower glucose metabolism in areas of the basal ganglia, including the right ventral striatum (Anthony et al., 2006) and caudate (Castellano et al., 2019) (cf.(Castellano et al., 2015)), but not with the left ventral striatum (Anthony et al., 2006). Higher IR were also associated with lower glucose metabolism in the amygdala (Castellano et al., 2015) (cf.(Anthony et al., 2006)), hippocampus (Castellano et al., 2015, Femminella et al., 2021), and thalamus (Castellano et al., 2019) (cf.(Castellano et al., 2015)). However, in one study, higher IR was associated with increased thalamic glucose metabolism (Chen et al., 2022).

*Note*. Given the large number of findings across brain areas, networks, and regions, results were grouped according to higher-order category where possible to give an indication of the most commonly reported areas showing significant responses to intranasal insulin administration.

### Experimental medicine models of brain insulin resistance: Intranasal insulin challenges

#### Magnetoencephalography

Stingl et al. (2010) found that intranasal insulin increases theta path length in lean subjects but decreases theta path length in obese subjects (Stingl et al., 2010). In other study by Guthoff et al. (2011), it was shown that intranasal insulin increases the ventral-stream M2 component (i.e., activity generated in higher visual areas, such as the fusiform gyrus) 140–190 ms after stimulus presentation in lean but not obese participants when viewing food stimuli (Guthoff et al., 2011). In the third study, intranasal insulin was shown to increase theta band power during a food-related working memory task in carriers of the cannabinoid-receptor 2 gene single nucleotide polymorphism (i.e., rs3123554)(Ketterer et al., 2014).

#### Neurotransmitters

In one study, intranasal insulin increased the binding potential of C11 raclopride, which binds to D2 and D3 receptors in the ventral and dorsal striatum, reflecting lower synaptic dopamine on PET (Kullmann et al., 2021).

#### Resting-state functional brain activity

**Striatal activity:** Two studies have found that intranasal insulin decreases activity in the caudate of the dorsal striatum (Heni et al., 2016, Kullmann et al., 2021). However, these effects seem to have differential outcomes depending on participants’ genome. In the study by Heni et al., (2016), participants were recruited on the basis of the D2 receptor allele (ANKK1 rs1800497 XX), which has been implicated in obesity risk (Heni et al., 2016). In people with this D2 receptor risk gene, insulin decreased caudate activity in people who carried another non-risk gene (FTO CC) but increased activity in people who were carriers of another obesity risk allele (FTO rs8050136 XA). In D2 receptor non-risk allele carriers, caudate activity seems to decrease but there was no difference in caudate activity across FTO risk and non-risk carriers.

**Hippocampal activity:** Across two studies, intranasal insulin increased connectivity beween the hippocampus and the medial prefrontal cortex(Kullmann et al., 2017; Zhang et al., 2015), as well as other regions of the DMN, including the posterior and anterior cingulate cortex(Zhang et al., 2015).

**Hypothalamic activity:** One study reported that intranasal insulin increased connectivity between the anterior medial prefrontal cortex and the hypothalamus in people with low peripheral IR (Kullmann et al., 2017). However, another study found that intranasal insulin reduces resting state activity in the left hypthalamus and left orbitofrontal cortex (Kullmann et al., 2013).

**Mesocorticolimbic pathways:** One study found a three-way interaction between dose, time, and peripheral insulin sensitivity on resting-state functional brain connectivity (Edwin Thanarajah et al., 2019). 40 IU and 160 IU increased connectivity between the ventral tegmental area and ventromedial prefrontal cortex 25–35 minutes after administration in participants with low peripheral IR. Compared to placebo, 40 IU and 100 IU increased connectivity between the ventral tegmental area and ventromedial prefrontal cortex 80–90 minutes after administration in people with high peripheral IR. 100 IU decreased connectivity between the ventral tegmental area and ventromedial prefrontal cortex in participants with higher peripheral IR.

#### Task-based functional brain activity

**Visual food cues:** When viewing food cues, intranasal insulin lead to decreased activity in the nucleus accumbens and left ventral tegmental area in people with low peripheral IR, but increased activity in people with high peripheral IR (Tiedemann et al., 2017). In another study, intranasal insulin led to increase amygdala activity when viewing high calorie foods (Wagner et al., 2022). In this same study, insulin also increased precuneus and insula activity in healthy males and overweight females. These same regions decreased in response to insulin in healthy females and overweight males. A similar pattern was seen in the dorsolateral prefrontal cortex, which increased with insulin in females and decreased in men when viewing highly desired food. In another study, it was found that intranasal insulin increased activity in the anterior cingulate cortex and nucleus accumbens to sugar cues in healthy controls more than in overweight participants (Wingrove et al., 2022). In another study, while insulin also reduced activity in the bilateral fusiform gyri, right hippocampus, right temporal superior cortex, and right middle frontal cortex compared to baseline, this increase was not different from placebo (Guthoff et al., 2010). When consuming sugar rather than viewing it, there was evidence that intranasal insulin leads to greater decreases in hypothalamic activity (Opstal et al., 2017), as well as greater decreases in amygdala activity in healthy controls compared to overweight participants (Wingrove et al., 2022).

**Sensory maze tasks:** A study by Brunner and colleagues (2016) found no evidence that intranasal insulin affected brain activity during visual or smell-based maze tasks (Brunner et al., 2016).

#### Cerebral blood flow dynamics

**Whole brain:** As published literature on whole-brain changes in cerebral blood flow (CBF) have tended to report null findings (Akintola et al., 2017, Kleinloog et al., 2022, Wingrove et al., 2021), there is little evidence that region-nonspecific changes in blood flow in the brain respond to intranasal insulin.

**Frontal lobe:** Four studies reported effects in the frontal lobe in response to intranasal insulin, finding increased CBF in the opercular part of the bilateral inferior frontal gyrus (Schilling et al., 2014) and medial prefrontal cortex (Novak et al., 2022), while reducing CBF in the right middle frontal gyrus (Kullmann et al., 2015). In one study, no changes in frontal lobe CBF were found (Akintola et al., 2017).

**Temporal lobe**. Four studies reported effects in the temporal lobe in response to intranasal insulin, finding increased blood flow in the bilateral insular cortex (Schilling et al., 2014) and right insula (Novak et al., 2014), but reduced CBF in the bilateral parahippocampal gyri, right fusiform gyrus, and left insula (Wingrove et al., 2021). In one study no changes in temporal lobe CBF were found (Akintola et al., 2017).

**Parietal lobe:** In one study, intranasal insulin resulted in decreased cerebrovascular reactivity in the posterior cingulate cortex and precuneus of the DMN (Novak et al., 2022).

**Occipital lobe:** Findings for an effect of intranasal insulin on cerebral blood flow in the occipital cortex have been mixed as one study found increased cerebral blood flow in the occipital lobe (Akintola et al., 2017) with another study reporting a null finding (Grichisch et al., 2012)

**Subcortical structures:** Five studies reported subcortical changes in response to intranasal insulin, finding increased CBF in the thalamus (Akintola et al., 2017), bilateral putamen, and left caudate (Schilling et al., 2014). However, in other regions reduced CBF has been reported, including in the hypothalamus (Kullmann et al., 2015) (cf.(Akintola et al., 2017)), bilateral amygdala (Wingrove et al., 2019), left putamen, and left hippocampus (Wingrove et al., 2021).

#### Brain glucose metabolism

There is limited evidence from one study that intranasal insulin has an effect on longitudinal changes in brain glucose metabolism. In a study by Craft et al. (2012), it was found that 20 IU and 40 IU of intranasal insulin reduced the progression of glucose hypometabolism in bilateral frontal, right temporal, and bilateral occipital lobes, as well as in the right precuneus. 20 IU, but not 40 IU, was also associated with reduced progression of hypometabolism in the right temporal lobe, whilst 40 IU was associated with reduced progression of hypometabolism in the left parietal cortex.

### Neuronally Enriched Extracellular Vesicles

Ten studies quantified proteins involved in insulin signalling in blood-derived exosomes released from cells that were enriched for neuronal origin. Of these, four studies assessed insulin signalling proteins in response to insulin sensitising drugs and six studies compared insulin signalling proteins between healthy controls and patients with disorders theoretically thought to affect brain insulin sensitivity, such as dementia, depression, and schizophrenia.

**Overall Insulin Signalling Pathway.** In one study, patients with disorders thought to result in brain IR (i.e., schizophrenia) had decreased phosphorylated to total protein ratios compared to healthy controls (Kapogiannis, Dobrowolny, et al., 2019).

**Upstream First Node.** Across three studies, there was evidence that treatment with drugs known to have insulin sensitising properties resulted in increased concentrations of pIGF-1R (Avgerinos et al., 2022) and pY-IRS-1(Athauda et al., 2019; Avgerinos et al., 2022) (cf. (Kapogiannis, Mustapic, et al., 2019)). Complimenting this finding, two other studies also found that patients with disorders thought to implicate brain IR (i.e., Parkinson’s disease with cognitive impairment and T2DM) had decreased levels of pY-IRS-1 (Blommer et al., 2022, Kapogiannis et al., 2015). However, in some studies disorders in which brain IR is thought to play a role (i.e., Alzheimer’s disease) also had higher levels of pY-IRS-1 compared to healthy controls (Kapogiannis, Mustapic, et al., 2019) (cf. (Blommer et al., 2022; Kapogiannis, Dobrowolny, et al., 2019)). As for pSer312-IRS-1, one study suggested that insulin sensitising drugs increase concentrations of this protein (Athauda et al., 2019) (cf. (Avgerinos et al., 2022; Kapogiannis, Mustapic, et al., 2019)), but disorders thought to implicate brain IR (i.e., Alzheimer’s disease and T2DM) also demonstrated higher concentrations of this protein in two other studies (Kapogiannis et al., 2015, Kapogiannis et al., 2019) (cf. (Blommer et al., 2022; Kapogiannis, Dobrowolny, et al., 2019)). As for the ratio of pSer312-IRS-1 to pY-IRS-1, one study suggested that insulin sensitising drugs decrease the ratio between the two proteins(Avgerinos et al., 2022), while disorders implicating brain IR, such as Alzheimer’s disease, T2DM, and Parkinson’s disease with cognitive impairment, increase ratios compared to healthy controls (Blommer et al., 2022, Kapogiannis et al., 2015) (cf. (Singh et al., 2022)). And finally, higher concentrations of pSer616-IRS-1(Athauda et al., 2019) (cf. (Kapogiannis, Mustapic, et al., 2019)) and pIR(Avgerinos et al., 2022), as well as IRS-1 (Kapogiannis et al., 2015, Kapogiannis et al., 2019), were also detected in response to insulin sensitising drugs, as well as in disorders though to implicate brain IR (i.e., Alzheimer’s disease).

**Canonical Pathway.** Treatment with drugs with insulin sensitising properties resulted in increased concentrations of total Akt (Athauda et al., 2019) (cf. (Avgerinos et al., 2022; Kapogiannis, Mustapic, et al., 2019)) and phosphorylated Akt (Athauda et al., 2019, Avgerinos et al., 2022, Kapogiannis et al., 2019). However, in another two studies insulin sensitising drugs did not change levels of t-GSK-3β, p-GSK-3β (Athauda et al., 2019, Kapogiannis et al., 2019), or factor scores for the canonical insulin signalling pathway (i.e., Akt, GSK-3B, p70S6K)(Mansur et al., 2021). Compared to healthy controls, there was no evidence that patients with disorders thought to result in brain IR (i.e., major depressive disorder and schizophrenia) had any differences in downstream insulin signalling proteins, such as Akt, GSK3B, or p70S6K(Singh et al., 2022; Wijtenburg et al., 2019).

**Alternative Pathway**. Across two studies, there was evidence that insulin sensitising drugs may increase concentrations of pp38 (Avgerinos et al., 2022, Mansur et al., 2021) (cf. (Athauda et al., 2019; Kapogiannis, Mustapic, et al., 2019)), pERK1/2 (Mansur et al., 2021) (cf. (Athauda et al., 2019; Avgerinos et al., 2022; Kapogiannis, Mustapic, et al., 2019)), and pJNK (Mansur et al., 2021) (cf. (Athauda et al., 2019; Kapogiannis, Mustapic, et al., 2019)). However, in one study, drugs known to have insulin sensitising properties also resulted in decreased concentrations of pJNK (Avgerinos et al., 2022) (cf. (Kapogiannis, Mustapic, et al., 2019)), whilst other research has found no change in t-p38 following the administration of insulin sensitising drugs (Athauda et al., 2019, Kapogiannis et al., 2019).

**mTOR pathway**. Across two studies, treatment with insulin sensitising drugs increased concentrations of p-mTOR (Athauda et al., 2019) (cf. (Kapogiannis, Mustapic, et al., 2019)) and pS6RP (Kapogiannis, Mustapic, et al., 2019), while having no effect on levels of t-mTOR (Athauda et al., 2019, Kapogiannis et al., 2019). Compared to healthy controls, patients with disorders thought to result in brain IR had decreased ratios of t-mTOR to p-mTOR (i.e., schizophrenia)(Kapogiannis, Dobrowolny, et al., 2019), but no differences in mTOR or p-mTOR in patients with Parkinson’s disease (Blommer et al., 2022).

## Discussion

The aim of the current review was to synthesise literature using in vivo methods that could be used as biomarkers for brain-specific IR in humans. Unlike well-defined and validated measures of systemic insulin sensitivity, which are based on impaired insulin-mediated uptake of glucose, an analogous gold-standard biomarker for in vivo brain-specific insulin sensitivity in humans is less established. That said, this review has outlined three common neuroimaging and extracellular vesicle approaches to capturing in vivo insulin sensitivity in the human central nervous system. Here, we reviewed these three biomarkers, including studies measuring (i) functional brain correlates of peripheral IR, (ii) functional brain responses to the administration of intranasal insulin, and (iii) concentrations of insulin signalling proteins in blood-derived extracellular vesicles that have been enriched for neuronal origin (mainly through L1CAM immunoprecipitation).

Across the neuroimaging modalities in the reviewed studies, we did not find clear evidence of a single region or network that consistently associated with levels of peripheral IR (as measured by the HEC technique, HOMA-IR, or various forms of glucose tolerance tests) or responded to the administration of intranasal insulin. Although there was some cross-over in the brain correlates of peripheral IR and the brain’s responsiveness to intranasal insulin, which will be discussed below, we found evidence that peripheral IR and intranasal insulin associated with changes in brain activity across many brain regions. This is not surprising given the widespread action and function of insulin in the brain. We will first propose some reasons why the brain correlates of peripheral IR and the brain’s responsiveness to intranasal insulin may differ, before tentatively suggesting that a detailed investigation of the default mode network (DMN) using these two methods in a single design may be a useful next step for establishing an in vivo biomarker of brain-specific IR in humans. It is argued that while the brain regions of this network had the most growing evidence of a correlation with peripheral IR and responsiveness to intranasal insulin, other areas should be considered. An investigation of the correlation between peripheral IR, the effect of intranasal insulin, imaging measures of the DMN, and the correlation with blood-based assays of brain IR is warranted.

### Possible explanations for diverse brain responses across correlational and experimental methods

The first reason for variability across the brain regions correlating with peripheral IR and the brain regions responding to intranasal insulin may be explained by post-mortem studies of patients with Alzheimer’s disease (AD), as well as animal models of AD, which have shown molecular pathology consistent with IR in the brain in the absence of more widespread systemic IR (Talbot et al., 2012). This suggests that studies finding correlations between brain function and peripheral IR do not necessarily entail IR within the brain itself. Rather, such studies could be capturing the brain’s adaptation to systemic IR in which the central nervous system has, nonetheless, maintained its sensitivity to insulin. Indeed, it is often assumed in the literature that brain IR is a consequence of the deleterious effects that systemic IR has on the central nervous system, including glucotoxicity, inflammation, and impaired cardiovascular functioning. In addition to evidence that central insulin action regulates both brain and body functions, the results of our review also demonstrate that brain-specific IR and the brain’s correlates of systemic IR can be separable, necessitating a brain-specific biomarker of IR (Rahul Agrawal et al., 2021).

The second reason for variability across the brain regions correlating with peripheral IR and the brain regions responding to intranasal insulin could lie in the fact that the insulin receptor gene differentially splices into long and short isoforms of the receptor, which are distributed differently throughout the body. The long isoform, known as insulin receptor B (IR-B), preponderates in the body’s adipose, renal, muscular, and hepatic tissues, playing a role in insulin’s metabolic effects in the periphery. Within the central nervous system, which was previously thought to be insensitive to insulin, IR-B, along with the short isoform, known as insulin receptor A (IR-A), are expressed across many brain regions. However, whilst glial cells, such as astrocytes, express both isoforms, neurons only express the short isoform of the insulin receptor (in contrast to the expression of the long isoform in the periphery). As such, there is precedence to look at the possibility of a distinction between impaired insulin sensitivity in the neurons of the central nervous system and the cells of the body’s peripheral tissues. For example, our review showed that whilst higher systemic IR is related to lower glucose uptake in the body’s peripheral organs, higher systemic IR is related to increased glucose metabolism in the brain, demonstrating the need to consider brain-specific IR as potentially distinct from more widespread insensitivity to insulin.

The third potential source for the observed variability across the brain regions correlating with peripheral IR and the brain regions responding to intranasal insulin could be due to the tools measuring systemic IR, such as the HEC technique and HOMA-IR, underestimating the level of IR in the body’s peripheral organs. Although the HEC technique is considered the gold standard, it comes with two problems when trying to probe brain-specific IR. First, the technique infuses high levels of insulin and glucose to artificially restrain insulin secretion from the pancreas and glucose uptake in the liver. Thus, measurements of IR with the HEC technique are perhaps not reflective of true physiological states, yielding different estimates of IR depending on the infusion rates used. Second, glucose uptake in the brain occurs at a constant rate and for the most part is not mediated by the action of insulin. This is not accounted for in the HEC technique’s estimates of IR, underestimating the true rates of IR in the body’s peripheral organs. As for HOMA-IR, estimates of IR are again reflective of non-physiological states. HOMA-IR estimates IR based on the balance between fasting glucose and insulin levels, which is regulated in a feedback loop between hepatic processes and pancreatic beta cells. In non-fasting states, however, the ingestion of food also leads to the secretion of incretin hormones, such as glucagon-like peptide 1 (GLP-1) and glucose-dependent insulinotropic polypeptide (GIP). Such hormones contribute to the glucose-induced secretion of insulin, thereby influencing measurements of IR in non-fasted physiological states but not in basal states, such as when conducting HOMA-IR. Together, these methods underestimate IR in the body’s peripheral organs, which would affect the associations with neuroimaging brain responses in the current review’s studies attempting to capture brain-specific IR by correlating system-wide IR with neuroimaging data.

The fourth potential reason is that whilst the definition of systemic IR is based on a reduction in insulin-mediated glucose uptake, the definition of brain-specific IR changes as a function of the in vivo neuroimaging modality used in any given study. According to our review, for example, brain IR could be defined as increased low-frequency fluctuations on resting-state fMRI in brain regions involved in saliency detection (Ryan et al., 2012), smaller reductions in blood-flow in the right middle frontal gyrus on ASL imaging (Kullmann et al., 2015), or smaller increases in the path length of theta frequencies on MEG in response to intranasal insulin (Stingl et al., 2010). Each of these in vivo methods captures different features of underlying brain function originating from different cell types: ASL captures cerebral blood flow (Petcharunpaisan et al., 2010), fMRI captures a combination of cerebral blood flow and oxygen extraction in neurons and astrocytes (Hillman, 2014), and MEG captures ionic currents during axonal propagation on the level of neurons only (Lopes da Silva, 2013). As few studies have taken a multimodal approach, in the absence of a ground truth for a singular definition of neuroimaging-based brain IR, it is difficult to ascertain if these varied approaches reflect the same neurophysiological processes.

### Using the default mode network as a biomarker of brain-specific insulin resistance

Having reviewed three existing biomarkers that could be used to define brain-specific IR, there seems to be no clear evidence of a single region or network that consistently associated across each study with levels of peripheral IR and responded to the administration of intranasal insulin. This is perhaps not surprising given insulin’s likely role in metabolic control, reward, and cognition. Indeed, insulin also serves as a satiety signal and provides a negative feedback signal to the brain, perhaps explaining why insulin may result in both increased and decreased brain activity across brain regions. There is also likely variability in brain responses to insulin due to variability in the insulin receptor and density, as well as signalling characteristics across brain regions. Nonetheless, there was a network of brain regions that correlated with systemic IR and responded to intranasal insulin, potentially pointing to a tentative biomarker of neuroimaging-based brain-specific IR. This network was the default mode network (DMN), which is a collection of brain structures that are preferentially activated at rest and deactivated when individuals are involved in cognitively demanding tasks (Raichle, 2015). Three of the main hubs of the DMN are the posterior cingulate cortex (PCC), precuneus, and hippocampus.

Notwithstanding the fact that the DMN is the most studied network in resting state neuroimaging, in our review, we reported evidence that higher systemic IR was related to lower connectivity in the PCC and precuneus (Cui et al., 2015, Musen et al., 2012), as well as connectivity between the PCC and other gyri across the temporal, occipital and frontal lobes (Chen et al., 2014). In one intranasal insulin study, the administration of insulin also resulted in increased connectivity between the hippocampus and the PCC (Zhang et al., 2015). In our review, we showed evidence that higher systemic IR was related to lower activity in the precuneus during working memory tasks (Williams et al., 2019), as well as reduced blood flow to the PCC and precuneus at rest (Cui et al., 2017). When viewing both high-calorie and low-calorie foods, higher IR was associated with higher activity in the PCC and precuneus (Van Vugt et al., 2014). There was also evidence that higher systemic IR related to lower glucose metabolism in the PCC and precuneus (Baker et al., 2011) in which one intranasal study showed that the administration of insulin slowed the rate of hypometabolism in the precuneus (Craft et al., 2012). In light of existing literature on Alzheimer’s disease, the DMN is a candidate for further exploration in defining in vivo brain-specific IR since the PCC shows early hypometabolism in AD (Valla et al., 2001), as well as early depositions of amyloid beta (Ali et al., 2022), which predicts the progression of cortical atrophy in AD and conversion of MCI to AD (Bailly et al., 2015, Kato et al., 2016). The precuneus has also been suggested as a key centre of early amyloid accumulation, showing depositions of beta amyloid even before conversion from normal to abnormal cerebrospinal fluid markers of the biomarker (Palmqvist et al., 2017). Disturbances in precuneus deactivation are then thought to reflect localised atrophy and research has suggested that this localised atrophy may precede regional atrophy as AD progresses, potentially tracking the progression of mild cognitive impairment to Alzheimer’s disease (Koch et al., 2022).

While it is acknowledged and expected that peripheral IR will correlate with many brain regions and that intranasal insulin administration will result in widespread changes in brain function, establishing an in vivo measure of brain-specific IR in humans requires evidence of a consistent brain correlate of brain responses. Compared to systemic measures of IR, which are defined with respect to glucose, our investigation sought to establish preliminary evidence of brain function and brain responses that could be analogous to the use of glucose in brain-specific IR. Although peripheral IR correlated with brain activity across many regions and modalities, and intranasal insulin resulted in widespread responsiveness in the brain, the DMN had the largest body of literature showing somewhat consistent responses. We emphasise that further investigation is needed, and the use of other brain proxies is not ruled out, but that the DMN may be a useful network for further exploration in establishing a brain-specific measure of in vivo IR in humans.

### Evidence of brain insulin resistance in blood derived extracellular vesicles

One approach to bypassing issues in varying neuroimaging modalities and peripheral IR measures, as outlined above, has been to quantify levels of proteins in extracellular vesicles that have been enriched for neuronal origin (with L1CAM). In this review, we included papers assessing protein levels in patients with assumed brain IR compared to healthy controls and papers assessing the effect of insulin sensitising drugs on the levels of proteins involved in the insulin signalling pathway. Across the included studies, differences in protein concentrations in response to insulin-sensitising drugs and in patients with disorders presumably implicating brain IR were mixed and often not consistent. That said, one of the more consistent findings was that disorders thought to implicate brain IR had increased levels of pY-IRS-1 and that insulin-sensitising drugs tended to decrease levels of this phosphorylated form of the insulin receptor substrate. A problem in this research, however, is the extent to which the ground truth that these disorders thought to implicate brain IR did indeed entail that patients had brain-specific IR. Without such knowledge, it is difficult to interpret the findings or hypothesise a priori differences in protein levels. Indeed, it should also be noted that precipitation of L1CAM is thought to enrich for neuronal origin, but this method of immunoprecipitation may not be central nervous system specific, perhaps suggesting the need for studies using cerebral spinal fluid to validate the findings. There is also growing evidence that extracellular vesicles can be used to measure glial and endothelial IR, which although not included in this review, may warrant exploration as these cells can be resistant to IR (Kumar et al., 2024). Of the papers included in this review on neuronally derived extracellular vesicles, it is noteworthy that no studies associated neuroimaging brain responses to intranasal insulin or brain correlates of systemic IR with levels of proteins in the neuronally-enriched extracellular vesicles. This is one avenue for identifying convergence in the biomarkers of brain IR by corroborating findings across blood biomarkers and neuroimaging responses.

### Suggestions for future research

To further efforts in defining an in vivo measure of brain-specific IR in humans, future research could focus on integrating neuroimaging and molecular measures to help establish an in vivo biomarker that is analogous to system-wide IR (which is tightly defined by glucose-insulin interactions). One promising approach could be to investigate the relationship between peripheral IR and brain activity within the default mode network (DMN) using resting-state MRI (rs-fMRI) to look at functional connectivity and arterial spin labelling (ASL) imaging to look at regional cerebral blood flow. To establish causality, the same measures could be used to assess changes in DMN activity in response to intranasal insulin administration in the same study with the same participants. In parallel, molecular-level validation of brain-specific IR could be explored by correlating these neuroimaging patterns in the DMN from the first two approaches with concentrations of phosphorylated insulin receptor substrate-1 at tyrosine sites (pY-IRS-1) in neuronally enriched extracellular vesicles (EVs) isolated from blood using L1CAM antibodies. By integrating neuroimaging and molecular markers in the same study, this approach has the potential to establish a robust, brain-specific measure of IR that is as well-defined as the systemic model based on glucose and insulin homeostasis. To date, and to the authors’ knowledge, no study has taken this approach, which could help establish an in vivo measure of brain-specific IR that is as clearly defined as system-wide IR. Establishing an in vivo biomarker of brain-specific IR in humans has clinical utility that could facilitate early detection and targeted interventions for conditions, such as Alzheimer's disease, where brain IR is thought to be implicated.

## Conclusion

While this review has uncovered no singular biomarker for in vivo brain-specific IR in humans, having a method of identifying people with brain IR is of clear importance in dementia prevention. In post-mortem studies of AD, hippocampal neurons seem to be less responsive to insulin stimulation and abnormal insulin signalling has been associated with higher levels of amyloid beta and tau, as well as impaired cognition. There is even some evidence that intranasal insulin may maintain cognitive abilities or reduce cognitive decline. Clinically, mild cognitive impairment (MCI) is a heterogenous group in which not all patients can be considered pre-AD. Thus, the ability to identify people at higher risk for AD who may have brain-specific IR would be important. However, the field needs a clear biomarker for identifying people at risk for brain-specific IR and then assessing their responsiveness to any insulin sensitising treatment in the brain. The results of our review tentatively suggest that a promising area of research would be the default mode network in which functional brain responses to intranasal insulin, as well as associations with systemic IR, may be a promising avenue for defining brain-specific IR in humans. This was suggested because although peripheral IR correlated with widespread brain activity across the brain and many brain regions responded to intranasal insulin in this review, the default mode network was one of the few networks with a growing number of papers suggesting consistent associations with peripheral IR and responses to intranasal administration of insulin. We do not suggest that the default mode network is the only useful biomarker, but rather that it may represent the strongest avenue to explore given the relative strength of evidence compared to other brain regions. Given no research has taken a multimodal neuroimaging approach, nor used complementary blood biomarkers of insulin sensitivity in a single design to capture brain-specific IR, we forward this suggestion for future research. Specifically, blood flow and resting state measures of brain connectivity that correlate with peripheral IR and respond to intranasal insulin, in addition to concentrations of pY-IRS-1, may be a promising multimodal approach to establish a scalable brain-specific measure of in vivo IR in humans if explored in a single design with the same participants.

## Acknowledgements

GR has received funding to support his work from Fives Lives and a Medical Research Council (MCR) Grant to Dementia Platform UK (DPUK; MR/T033371/1). SB declares funding from DPUK in support of her work. AA declares funding from the 10.13039/501100013373NIHR Oxford Biomedical Research Centre (BRC). IK declares funding through DPUK (MCR), NIHR Oxford Health Biomedical Research Centre, and NIHR personal awards. This research was funded in whole, or in part, by the 10.13039/100014013UKRI [MR/T033371/1]. For the purpose of open access, the author has applied a CC BY public copyright licence to any author accepted manuscript version arising from this submission.

## Author Contributions

GR, SB, AA, and IK contributed on the conceptualisation of the review. GR and BS reviewed the relevant literature for the manuscript. GR wrote the original draft and all authors contributed to the reviewing and editing of the manuscript.

## CRediT authorship contribution statement

**Adler Amanda:** Writing – review & editing, Supervision, Conceptualization. **Bauermeister Sarah:** Writing – review & editing, Supervision, Conceptualization. **Sargent Brendan:** Writing – review & editing, Validation, Investigation, Formal analysis, Data curation. **Reid Graham:** Writing – review & editing, Writing – original draft, Methodology, Investigation, Formal analysis, Data curation, Conceptualization. **Koychev Ivan:** Writing – review & editing, Supervision, Project administration, Conceptualization.

## Declaration of Competing Interest

The authors declare that they have no known competing financial interests or personal relationships that could have appeared to influence the work reported in this paper.

## References

[bib1] Adamo M., Raizada M.K., LeRoith D. (1989). Insulin and insulin-like growth factor receptors in the nervous system. Mol. Neurobiol..

[bib2] Adzovic L., Lynn A.E., D’Angelo H.M., Crockett A.M., Kaercher R.M., Royer S.E., Hopp S.C., Wenk G.L. (2015). Insulin improves memory and reduces chronic neuroinflammation in the hippocampus of young but not aged brains. J. Neuroinflamm..

[bib3] Agostinone J., Alarcon-Martinez L., Gamlin C., Yu W.-Q., Wong R.O.L., Di Polo A. (2018). Insulin signalling promotes dendrite and synapse regeneration and restores circuit function after axonal injury. Brain.

[bib4] Akin O., Eker I., Arslan M., Yavuz S.T., Akman S., Tascilar M.E., Unay B. (2017). Relation of insulin resistance to neurocognitive function and electroencephalography in obese children. J. Pediatr. Endocrinol. Metab..

[bib5] Akintola A.A., van Opstal A.M., Westendorp R.G., Postmus I., van der Grond J., van Heemst D. (2017). Effect of intranasally administered insulin on cerebral blood flow and perfusion; a randomized experiment in young and older adults. Aging.

[bib6] Albai O., Frandes M., Timar R., Roman D., Timar B. (2019). Risk factors for developing dementia in type 2 diabetes mellitus patients with mild cognitive impairment. Neuropsychiatr. Dis. Treat..

[bib7] Ali D.G., Bahrani A.A., Barber J.M., El Khouli R.H., Gold B.T., Harp J.P., Jiang Y., Wilcock D.M., Jicha G.A. (2022). Amyloid-PET Levels in the Precuneus and Posterior Cingulate Cortices Are Associated with Executive Function Scores in Preclinical Alzheimer's Disease Prior to Overt Global Amyloid Positivity. J. Alzheimer'S. Dis..

[bib8] Alsaadi H.M., Van Vugt D.A. (2015). Insulin sensitivity affects corticolimbic brain responses to visual food cues in polycystic ovary syndrome patients. Horm. Mol. Biol. Clin. Investig..

[bib9] Anthony K., Reed L.J., Dunn J.T., Bingham E., Hopkins D., Marsden P.K., Amiel S.A. (2006). Attenuation of insulin-evoked responses in brain networks controlling appetite and reward in insulin resistance: the cerebral basis for impaired control of food intake in metabolic syndrome?. Diabetes.

[bib10] Arvanitakis Z., Wang H.-Y., Capuano A.W., Khan A., Taïb B., Anokye-Danso F., Schneider J.A., Bennett D.A., Ahima R.S., Arnold S.E. (2020). Brain Insulin Signaling, Alzheimer Disease Pathology, and Cognitive Function. Ann. Neurol..

[bib11] Athauda D., Gulyani S., Karnati H.K., Li Y., Tweedie D., Mustapic M., Chawla S., Chowdhury K., Skene S.S., Greig N.H., Kapogiannis D., Foltynie T. (2019). Utility of Neuronal-Derived Exosomes to Examine Molecular Mechanisms That Affect Motor Function in Patients With Parkinson Disease: A Secondary Analysis of the Exenatide-PD Trial. JAMA Neurol..

[bib12] Avgerinos K.I., Mullins R.J., Vreones M., Mustapic M., Chen Q., Melvin D., Kapogiannis D., Egan J.M. (2022). Empagliflozin Induced Ketosis, Upregulated IGF-1/Insulin Receptors and the Canonical Insulin Signaling Pathway in Neurons, and Decreased the Excitatory Neurotransmitter Glutamate in the Brain of Non-Diabetics. Cells.

[bib13] Bahniwal M., Little J.P., Klegeris A. (2017). High Glucose Enhances Neurotoxicity and Inflammatory Cytokine Secretion by Stimulated Human Astrocytes. Curr. Alzheimer Res.

[bib14] Bailly M., Destrieux C., Hommet C., Mondon K., Cottier J.-P., Beaufils E., Vierron E., Vercouillie J., Ibazizene M., Voisin T., Payoux P., Barré L., Camus V., Guilloteau D., Ribeiro M.-J. (2015). Precuneus and Cingulate Cortex Atrophy and Hypometabolism in Patients with Alzheimer’s Disease and Mild Cognitive Impairment: MRI and 18F-FDG PET Quantitative Analysis Using FreeSurfer. BioMed. Res. Int..

[bib15] Baker L.D., Cross D.J., Minoshima S., Belongia D., Watson G.S., Craft S. (2011). Insulin resistance and Alzheimer-like reductions in regional cerebral glucose metabolism for cognitively normal adults with prediabetes or early type 2 diabetes. Arch. Neurol..

[bib16] Belfiore A., Malaguarnera R., Vella V., Lawrence M.C., Sciacca L., Frasca F., Morrione A., Vigneri R. (2017). Insulin Receptor Isoforms in Physiology and Disease: An Updated View. Endocr. Rev..

[bib17] Beneit N., Fernández-García C.E., Martín-Ventura J.L., Perdomo L., Escribano Ó., Michel J.B., García-Gómez G., Fernández S., Díaz-Castroverde S., Egido J., Gómez-Hernández A., Benito M. (2016). Expression of insulin receptor (IR) A and B isoforms, IGF-IR, and IR/IGF-IR hybrid receptors in vascular smooth muscle cells and their role in cell migration in atherosclerosis. Cardiovasc. Diabetol..

[bib18] Blommer J., Pitcher T., Mustapic M., Eren E., Yao P.J., Vreones M.P., Pucha K.A., Dalrymple-Alford J., Shoorangiz R., Meissner W.G., Anderson T., Kapogiannis D. (2022). Extracellular vesicle biomarkers for cognitive impairment in Parkinson's disease. Brain.

[bib19] Boersma G.J., Johansson E., Pereira M.J., Heurling K., Skrtic S., Lau J., Katsogiannos P., Panagiotou G., Lubberink M., Kullberg J., Ahlstrom H., Eriksson J.W. (2018). Altered Glucose Uptake in Muscle, Visceral Adipose Tissue, and Brain Predict Whole-Body Insulin Resistance and may Contribute to the Development of Type 2 Diabetes: A Combined PET/MR Study. Horm. Metab. Res.

[bib20] Brunner Y.F., Rodriguez-Raecke R., Mutic S., Benedict C., Freiherr J. (2016). Neural correlates of olfactory and visual memory performance in 3D-simulated mazes after intranasal insulin application. Neurobiol. Learn. Mem..

[bib21] Castellano C.A., Baillargeon J.P., Nugent S., Tremblay S., Fortier M., Imbeault H., Duval J., Cunnane S.C. (2015). Regional Brain Glucose Hypometabolism in Young Women with Polycystic Ovary Syndrome: Possible Link to Mild Insulin Resistance. PLoS ONE [Electron. Resour. ].

[bib22] Castellano C.A., Hudon C., Croteau E., Fortier M., St-Pierre V., Vandenberghe C., Nugent S., Tremblay S., Paquet N., Lepage M., Fulop T., Turcotte E.E., Dionne I.J., Potvin O., Duchesne S., Cunnane S.C. (2019). Links Between Metabolic and Structural Changes in the Brain of Cognitively Normal Older Adults: A 4-Year Longitudinal Follow-Up. Front. Aging Neurosci..

[bib23] Chatterjee S., Ambegaokar S.S., Jackson G.R., Mudher A. (2019). Insulin-mediated changes in tau hyperphosphorylation and autophagy in a Drosophila model of tauopathy and neuroblastoma cells. Front. Neurosci..

[bib24] Chen Y., Qiu C., Yu W., Zhou M., Wang Y., Shao X. (2022). The relationship between brain glucose metabolism and insulin resistance in subjects with normal cognition - A study based on 18F-FDG PET. Nucl. Med. Commun..

[bib25] Chen Y.C., Jiao Y., Cui Y., Shang S.A., Ding J., Feng Y., Song W., Ju S.H., Teng G.J. (2014). Aberrant brain functional connectivity related to insulin resistance in type 2 diabetes: a resting-state fMRI study. Diabetes Care.

[bib26] Craft S., Baker L.D., Montine T.J., Minoshima S., Watson G.S., Claxton A., Arbuckle M., Callaghan M., Tsai E., Plymate S.R., Green P.S., Leverenz J., Cross D., Gerton B. (2012). Intranasal insulin therapy for Alzheimer disease and amnestic mild cognitive impairment: a pilot clinical trial. Arch. Neurol..

[bib27] Cui Y., Jiao Y., Chen H.J., Ding J., Luo B., Peng C.Y., Ju S.H., Teng G.J. (2015). Aberrant functional connectivity of default-mode network in type 2 diabetes patients. Eur. Radio..

[bib28] Cui Y., Liang X., Gu H., Hu Y., Zhao Z., Yang X.-Y., Qian C., Yang Y., Teng G.-J. (2017). Cerebral perfusion alterations in type 2 diabetes and its relation to insulin resistance and cognitive dysfunction. Brain Imaging Behav..

[bib29] Dai H., Zhang Y., Lai L., Hu S., Wang X., Li Y., Hu C., Shen H. (2017). Brain functional networks: correlation analysis with clinical indexes in patients with diabetic retinopathy. Neuroradiology.

[bib30] Devaskar S.U., Giddings S.J., Rajakumar P.A., Carnaghi L.R., Menon R.K., Zahm D.S. (1994). Insulin gene expression and insulin synthesis in mammalian neuronal cells. J. Biol. Chem..

[bib31] Dorn A., Rinne A., Bernstein H.G., Hahn H.J., Ziegler M. (1983). Insulin and C-peptide in human brain neurons (insulin/C-peptide/brain peptides/immunohistochemistry/radioimmunoassay). J. Hirnforsch..

[bib32] Drummen M., Dorenbos E., Vreugdenhil A.C.E., Raben A., Westerterp-Plantenga M.S., Adam T.C. (2019). Insulin resistance, weight, and behavioral variables as determinants of brain reactivity to food cues: a Prevention of Diabetes through Lifestyle Intervention and Population Studies in Europe and around the World - a PREVIEW study. Am. J. Clin. Nutr..

[bib33] Eckstrand K.L., Mummareddy N., Kang H., Cowan R., Zhou M., Zald D., Silver H.J., Niswender K.D., Avison M.J. (2017). An insulin resistance associated neural correlate of impulsivity in type 2 diabetes mellitus. PLoS ONE [Electron. Resour. ].

[bib34] Edwin Thanarajah S., Iglesias S., Kuzmanovic B., Rigoux L., Stephan K.E., Bruning J.C., Tittgemeyer M. (2019). Modulation of midbrain neurocircuitry by intranasal insulin. Neuroimage.

[bib35] Elizabeth M. Rhea M.L., Yassine Hussein N., Capuano Ana W., Tong Han, Petyuk Vladislav A., Macauley Shannon L., Fioramonti Xavier, Carmichael Owen, Calon Frederic, Arvanitakis Zoe (2023). State of the Science on Brain Insulin Resistance and Cognitive Decline Due to Alzheimer’s Disease. Aging Dis..

[bib36] Eriksson J.W., Visvanathar R., Kullberg J., Strand R., Skrtic S., Ekstrom S., Lubberink M., Lundqvist M.H., Katsogiannos P., Pereira M.J., Ahlstrom H. (2021). Tissue-specific glucose partitioning and fat content in prediabetes and type 2 diabetes: whole-body PET/MRI during hyperinsulinemia. Eur. J. Endocrinol..

[bib37] Femminella G.D., Livingston N.R., Raza S., van der Doef T., Frangou E., Love S., Busza G., Calsolaro V., Carver S., Holmes C., Ritchie C.W., Lawrence R.M., McFarlane B., Tadros G., Ridha B.H., Bannister C., Walker Z., Archer H., Coulthard E., Edison P. (2021). Does insulin resistance influence neurodegeneration in non-diabetic Alzheimer's subjects?. Alzheimer'S. Res. Ther..

[bib38] Garcia-Casares N., Berthier M.L., Jorge R.E., Gonzalez-Alegre P., Gutierrez Cardo A., Rioja Villodres J., Acion L., Ariza Corbo M.J., Nabrozidis A., Garcia-Arnes J.A., Gonzalez-Santos P. (2014). Structural and functional brain changes in middle-aged type 2 diabetic patients: a cross-sectional study. J. Alzheimer'S. Dis..

[bib39] Gastaldelli A. (2022). Measuring and estimating insulin resistance in clinical and research settings. Obesity.

[bib40] Gence L., Fernezelian D., Meilhac O., Rastegar S., Bascands J.L., Diotel N. (2023). Insulin signaling promotes neurogenesis in the brain of adult zebrafish. J. Comp. Neurol..

[bib41] Gonzales M.M., Tarumi T., Miles S.C., Tanaka H., Shah F., Haley A.P. (2010). Insulin sensitivity as a mediator of the relationship between BMI and working memory-related brain activation. Obesity.

[bib42] Grichisch Y., Cavusoglu M., Preissl H., Uludag K., Hallschmid M., Birbaumer N., Haring H.U., Fritsche A., Veit R. (2012). Differential effects of intranasal insulin and caffeine on cerebral blood flow. Hum. Brain Mapp..

[bib43] Gutch M., Kumar S., Razi S.M., Gupta K.K., Gupta A. (2015). Assessment of insulin sensitivity/resistance. Indian J. Endocrinol. Metab..

[bib44] Guthoff M., Grichisch Y., Canova C., Tschritter O., Veit R., Hallschmid M., Haring H.U., Preissl H., Hennige A.M., Fritsche A. (2010). Insulin modulates food-related activity in the central nervous system. J. Clin. Endocrinol. Metab..

[bib45] Guthoff M., Stingl K.T., Tschritter O., Rogic M., Heni M., Stingl K., Hallschmid M., Haring H.U., Fritsche A., Preissl H. (2011). The insulin-mediated modulation of visually evoked magnetic fields is reduced in obese subjects. PLoS ONE.

[bib46] He L., Vanlandewijck M., Mäe M.A., Andrae J., Ando K., Del Gaudio F., Nahar K., Lebouvier T., Laviña B., Gouveia L. (2018). Single-cell RNA sequencing of mouse brain and lung vascular and vessel-associated cell types. Sci. data.

[bib47] Heni M., Kullmann S., Ahlqvist E., Wagner R., Machicao F., Staiger H., Haring H.U., Almgren P., Groop L.C., Small D.M., Fritsche A., Preissl H. (2016). Interaction between the obesity-risk gene FTO and the dopamine D2 receptor gene ANKK1/TaqIA on insulin sensitivity. Diabetologia.

[bib48] Hillman E.M. (2014). Coupling mechanism and significance of the BOLD signal: a status report. Annu Rev. Neurosci..

[bib49] Honkala S.M., Johansson J., Motiani K.K., Eskelinen J.J., Virtanen K.A., Loyttyniemi E., Knuuti J., Nuutila P., Kalliokoski K.K., Hannukainen J.C. (2018). Short-term interval training alters brain glucose metabolism in subjects with insulin resistance. J. Cereb. Blood Flow. Metab..

[bib50] Hoscheidt S.M., Kellawan J.M., Berman S.E., Rivera-Rivera L.A., Krause R.A., Oh J.M., Beeri M.S., Rowley H.A., Wieben O., Carlsson C.M., Asthana S., Johnson S.C., Schrage W.G., Bendlin B.B. (2017). Insulin resistance is associated with lower arterial blood flow and reduced cortical perfusion in cognitively asymptomatic middle-aged adults. J. Cereb. Blood Flow. Metab..

[bib51] Ishibashi K., Onishi A., Fujiwara Y., Ishiwata K., Ishii K. (2017). Effects of glucose, insulin, and insulin resistance on cerebral18F-FDG distribution in cognitively normal older subjects. PLoS ONE.

[bib52] Johansson E., Lubberink M., Heurling K., Eriksson J.W., Skrtic S., Ahlstrom H., Kullberg J. (2018). Whole-Body Imaging of Tissue-specific Insulin Sensitivity and Body Composition by Using an Integrated PET/MR System: A Feasibility Study. Radiology.

[bib53] Julia P. Dunn R.M.K., Feurer Irene D., Volkow Nora D., Patterson Bruce W., Ansari Mohammad S., Li Rui, Marks-Shulman Pamela, Abumrad Naji N. (2012). Relationship of Dopamine Type 2 Receptor Binding Potential With Fasting Neuroendocrine Hormones and Insulin Sensitivity in Human Obesity. Diabetes Care.

[bib54] Kapogiannis D., Boxer A., Schwartz J.B., Abner E.L., Biragyn A., Masharani U., Frassetto L., Petersen R.C., Miller B.L., Goetzl E.J. (2015). Dysfunctionally phosphorylated type 1 insulin receptor substrate in neural-derived blood exosomes of preclinical Alzheimer's disease. FASEB J..

[bib55] Kapogiannis D., Dobrowolny H., Tran J., Mustapic M., Frodl T., Meyer-Lotz G., Schiltz K., Schanze D., Rietschel M., Bernstein H.G., Steiner J. (2019). Insulin-signaling abnormalities in drug-naive first-episode schizophrenia: Transduction protein analyses in extracellular vesicles of putative neuronal origin. Eur. Psychiatry.: J. Assoc. Eur. Psychiatr..

[bib56] Kapogiannis D., Mustapic M., Shardell M.D., Berkowitz S.T., Diehl T.C., Spangler R.D., Tran J., Lazaropoulos M.P., Chawla S., Gulyani S., Eitan E., An Y., Huang C.W., Oh E.S., Lyketsos C.G., Resnick S.M., Goetzl E.J., Ferrucci L. (2019). Association of Extracellular Vesicle Biomarkers With Alzheimer Disease in the Baltimore Longitudinal Study of Aging. JAMA Neurol..

[bib57] Kato T., Inui Y., Nakamura A., Ito K. (2016). Brain fluorodeoxyglucose (FDG) PET in dementia. Ageing Res. Rev..

[bib58] Kellar D., Craft S. (2020). Brain insulin resistance in Alzheimer's disease and related disorders: mechanisms and therapeutic approaches. Lancet Neurol..

[bib59] Ketterer C., Heni M., Stingl K., Tschritter O., Linder K., Wagner R., Machicao F., Haring H.U., Preissl H., Staiger H., Fritsche A. (2014). Polymorphism rs3123554 in CNR2 reveals gender-specific effects on body weight and affects loss of body weight and cerebral insulin action. Obesity.

[bib60] Kleinloog J.P.D., Mensink R.P., Smeets E., Ivanov D., Joris P.J. (2022). Acute inorganic nitrate intake increases regional insulin action in the brain: results of a double-blind, randomized, controlled cross-over trial with abdominally obese men. NeuroImage. Clin..

[bib61] Koch G., Casula E.P., Bonnì S., Borghi I., Assogna M., Minei M., Pellicciari M.C., Motta C., D’Acunto A., Porrazzini F., Maiella M., Ferrari C., Caltagirone C., Santarnecchi E., Bozzali M., Martorana A. (2022). Precuneus magnetic stimulation for Alzheimer’s disease: a randomized, sham-controlled trial. Brain.

[bib62] Kullmann S., Blum D., Jaghutriz B.A., Gassenmaier C., Bender B., Haring H.U., Reischl G., Preissl H., la Fougere C., Fritsche A., Reimold M., Heni M. (2021). Central Insulin Modulates Dopamine Signaling in the Human Striatum. J. Clin. Endocrinol. Metab..

[bib63] Kullmann S., Frank S., Heni M., Ketterer C., Veit R., Haring H.U., Fritsche A., Preissl H. (2013). Intranasal insulin modulates intrinsic reward and prefrontal circuitry of the human brain in lean women. Neuroendocrinology.

[bib64] Kullmann S., Heni M., Veit R., Ketterer C., Schick F., Haring H.U., Fritsche A., Preissl H. (2012). The obese brain: association of body mass index and insulin sensitivity with resting state network functional connectivity. Hum. Brain Mapp..

[bib65] Kullmann S., Heni M., Veit R., Scheffler K., Machann J., Häring H.-U., Fritsche A., Preissl H. (2015). Selective insulin resistance in homeostatic and cognitive control brain areas in overweight and obese adults. Diabetes Care.

[bib66] Kullmann S., Heni M., Veit R., Scheffler K., Machann J., Haring H.U., Fritsche A., Preissl H. (2017). Intranasal insulin enhances brain functional connectivity mediating the relationship between adiposity and subjective feeling of hunger. Sci. Rep..

[bib67] Kumar A., Nader M.A., Deep G. (2024). Emergence of Extracellular Vesicles as "Liquid Biopsy" for Neurological Disorders: Boom or Bust. Pharm. Rev..

[bib68] Kuwabara T., Kagalwala M.N., Onuma Y., Ito Y., Warashina M., Terashima K., Sanosaka T., Nakashima K., Gage F.H., Asashima M. (2011). Insulin biosynthesis in neuronal progenitors derived from adult hippocampus and the olfactory bulb. EMBO Mol. Med.

[bib69] Latva-Rasku A., Honka M.-J., Stancáková A., Koistinen H.A., Kuusisto J., Li G., Manning A.K., Stringham H., Gloyn A.L., Lindgren C.M., Collins F.S., Mohlke K.L., Scott L.J., Karjalainen T., Nummenmaa L., Boehnke M., Nuutila P., Laakso M., Stančáková A., Guan L. (2018). A Partial Loss-of-Function Variant in Is Associated With Reduced Insulin-Mediated Glucose Uptake in Multiple Insulin-Sensitive Tissues: A Genotype-Based Callback Positron Emission Tomography Study. Diabetes.

[bib70] Li M., Chi X., Wang Y., Setrerrahmane S., Xie W., Xu H. (2022). Trends in insulin resistance: insights into mechanisms and therapeutic strategy. Signal Transduct. Target. Ther..

[bib71] Livingston G., Huntley J., Sommerlad A., Ames D., Ballard C., Banerjee S., Brayne C., Burns A., Cohen-Mansfield J., Cooper C., Costafreda S.G., Dias A., Fox N., Gitlin L.N., Howard R., Kales H.C., Kivimäki M., Larson E.B., Ogunniyi A., Mukadam N. (2020). Dementia prevention, intervention, and care: 2020 report of the <em>Lancet< /em> Commission. Lancet.

[bib72] Long C., Han X., Yang Y., Li T., Zhou Q., Chen Q. (2022). Efficacy of intranasal insulin in improving cognition in mild cognitive impairment or dementia: a systematic review and meta-analysis. Front. Aging Neurosci..

[bib73] Lopes da Silva F. (2013). EEG and MEG: Relevance to Neuroscience. Neuron.

[bib74] Lopez-Vilaret K.M., Fernandez-Alvarez M., Shokri-Kojori E., Tomasi D., Cantero J.L., Atienza M. (2022). Pre-diabetes is associated with altered functional connectivity density in cortical regions of the default-mode network. Front Aging Neurosci..

[bib75] Mansur R.B., Delgado-Peraza F., Subramaniapillai M., Lee Y., Iacobucci M., Nasri F., Rodrigues N., Rosenblat J.D., Brietzke E., Cosgrove V.E., Kramer N.E., Suppes T., Raison C.L., Fagiolini A., Rasgon N., Chawla S., Nogueras-Ortiz C., Kapogiannis D., McIntyre R.S. (2021). Exploring brain insulin resistance in adults with bipolar depression using extracellular vesicles of neuronal origin. J. Psychiatr. Res..

[bib76] Mantych G.J., James D.E., Chung H.D., Devaskar S.U. (1992). Cellular localization and characterization of Glut 3 glucose transporter isoform in human brain. Endocrinology.

[bib77] Marder T.J., Flores V.L., Bolo N.R., Hoogenboom W.S., Simonson D.C., Jacobson A.M., Foote S.E., Shenton M.E., Sperling R.A., Musen G. (2014). Task-induced brain activity patterns in type 2 diabetes: a potential biomarker for cognitive decline. Diabetes.

[bib78] Marks J.L., Porte D., Stahl W.L., Baskin D.G. (1990). Localization of insulin receptor mRNA in rat brain by in situ hybridization. Endocrinology.

[bib79] Mazucanti C.H., Liu Q.R., Lang D., Huang N., O'Connell J.F., Camandola S., Egan J.M. (2019). Release of insulin produced by the choroid plexis is regulated by serotonergic signaling. JCI Insight.

[bib80] Mazza E., Calesella F., Paolini M., di Pasquasio C., Poletti S., Lorenzi C., Falini A., Zanardi R., Colombo C., Benedetti F. (2022). Insulin resistance disrupts white matter microstructure and amplitude of functional spontaneous activity in Bipolar disorder. Bipolar Disord..

[bib81] McEwen B.S., Reagan L.P. (2004). Glucose transporter expression in the central nervous system: relationship to synaptic function. Eur. J. Pharm..

[bib82] Milstein J.L., Ferris H.A. (2021). The brain as an insulin-sensitive metabolic organ. Mol. Metab..

[bib83] Molnár G., Faragó N., Kocsis Á.K., Rózsa M., Lovas S., Boldog E., Báldi R., Csajbók É ., Gardi J., Puskás L.G. (2014). GABAergic neurogliaform cells represent local sources of insulin in the cerebral cortex. J. Neurosci..

[bib84] Morgello S., Uson R.R., Schwartz E.J., Haber R.S. (1995). The human blood-brain barrier glucose transporter (GLUT1) is a glucose transporter of gray matter astrocytes. Glia.

[bib85] Musen G., Jacobson A.M., Bolo N.R., Simonson D.C., Shenton M.E., McCartney R.L., Flores V.L., Hoogenboom W.S. (2012). Resting-state brain functional connectivity is altered in type 2 diabetes. Diabetes.

[bib86] Nam H.Y., Jun S., Pak K., Kim I.J. (2017). Concurrent Low Brain and High Liver Uptake on FDG PET Are Associated with Cardiovascular Risk Factors. Korean J. Radio..

[bib87] Novak V., Mantzoros C.S., Novak P., McGlinchey R., Dai W., Lioutas V., Buss S., Fortier C.B., Khan F., Aponte Becerra L. (2022). MemAID: memory advancement with intranasal insulin vs. placebo in type 2 diabetes and control participants: a randomized clinical trial. J. Neurol..

[bib88] Novak V., Milberg W., Hao Y., Munshi M., Novak P., Galica A., Manor B., Roberson P., Craft S., Abduljalil A. (2014). Enhancement of vasoreactivity and cognition by intranasal insulin in type 2 diabetes. Diabetes Care.

[bib89] O'Neill C., Kiely A.P., Coakley M.F., Manning S., Long-Smith C.M. (2012). Insulin and IGF-1 signalling: longevity, protein homoeostasis and Alzheimer's disease. Biochem Soc. Trans..

[bib90] Opstal A.M.V., Akintola A.A., Elst M.V., Westendorp R.G., Pijl H., Heemst D.V., Grond J.V. (2017). Effects of intranasal insulin application on the hypothalamic BOLD response to glucose ingestion. Sci. Rep..

[bib91] Palmqvist S., Schöll M., Strandberg O., Mattsson N., Stomrud E., Zetterberg H., Blennow K., Landau S., Jagust W., Hansson O. (2017). Earliest accumulation of β-amyloid occurs within the default-mode network and concurrently affects brain connectivity. Nat. Commun..

[bib92] Pemberton S., Galindo D.C., Schwartz M.W., Banks W.A., Rhea E.M. (2022). Endocytosis of insulin at the blood-brain barrier [Original Research]. Front. Drug Deliv..

[bib93] Petcharunpaisan S., Ramalho J., Castillo M. (2010). Arterial spin labeling in neuroimaging. World J. Radio..

[bib94] Poessel M., Morys F., Breuer N., Villringer A., Hummel T., Horstmann A. (2022). Brain response to food odors is not associated with body mass index and obesity-related metabolic health measures. Appetite.

[bib95] Rahul Agrawal C.M.R., Sharma Sunny, Christensen Camille, Huang Yiqing, Fisher Simon J. (2021). Insulin action in the brain regulates both central and peripheral functions. Am. J. Physiol. Endocrinol. Metab..

[bib96] Raichle M.E. (2015). The Brain's Default Mode Network. Annu. Rev. Neurosci..

[bib97] Rebelos E., Nummenmaa L., Dadson P., Latva-Rasku A., Nuutila P. (2021). Brain insulin sensitivity is linked to body fat distribution-the positron emission tomography perspective. Eur. J. Nucl. Med. Mol. Imaging.

[bib98] Rodriguez-Flores M., Garcia-Garcia E., Cano-Nigenda C.V., Cantu-Brito C. (2014). Relationship of obesity and insulin resistance with the cerebrovascular reactivity: a case control study. Cardiovasc. Diabetol..

[bib99] Ryan J.P., Karim H.T., Aizenstein H.J., Helbling N.L., Toledo F.G.S. (2018). Insulin sensitivity predicts brain network connectivity following a meal. Neuroimage.

[bib100] Ryan J.P., Sheu L.K., Critchley H.D., Gianaros P.J. (2012). A neural circuitry linking insulin resistance to depressed mood. Psychosom. Med..

[bib101] Sarah D.B., Michael Ben Y., Graham R., Gregory H., Karen R., Tam W., Sarah G., Graciela Muniz T., Ivan K. (2023). Insulin resistance, age and depression’s impact on cognition in middle-aged adults from the PREVENT cohort. BMJ Ment. Health.

[bib102] Schilling, Ferreira de Sá T.M., S D., Westerhausen, Strelzyk R., Larra F., Hallschmid M.F., Savaskan M., Oitzl E., S M., Busch H.P., Naumann E. (2014). Intranasal insulin increases regional cerebral blood flow in the insular cortex in men independently of cortisol manipulation. Hum. brain Mapp..

[bib103] Shin J.H. (2022). Dementia Epidemiology Fact Sheet 2022. Ann. Rehabil. Med.

[bib104] Shypshyna M., Kolesnyk O., Fedulova S., Veselovsky N. (2023). Insulin modulates the paired-pulse plasticity at glutamatergic synapses of hippocampal neurons under hypoinsulinemia. Front. Cell. Neurosci..

[bib105] Singh D., Dobrowolny H., Kapogiannis D., Steiner J. (2022). Canonical insulin signaling is not significantly impaired in early stages of depression. Eur. Arch. Psychiatry Clin. Neurosci..

[bib106] Steen E., Terry B.M., Rivera E.J., Cannon J.L., Neely T.R., Tavares R., Xu X.J., Wands J.R., de la Monte S.M. (2005). Impaired insulin and insulin-like growth factor expression and signaling mechanisms in Alzheimer's disease--is this type 3 diabetes?. J. Alzheimer'S. Dis..

[bib107] Stingl K.T., Kullmann S., Guthoff M., Heni M., Fritsche A., Preissl H. (2010). Insulin modulation of magnetoencephalographic resting state dynamics in lean and obese subjects. Front. Syst. Neurosci..

[bib108] Talbot K., Wang H.-Y., Kazi H., Han L.-Y., Bakshi K.P., Stucky A., Fuino R.L., Kawaguchi K.R., Samoyedny A.J., Wilson R.S., Arvanitakis Z., Schneider J.A., Wolf B.A., Bennett D.A., Trojanowski J.Q., Arnold S.E. (2012). Demonstrated brain insulin resistance in Alzheimer’s disease patients is associated with IGF-1 resistance, IRS-1 dysregulation, and cognitive decline. J. Clin. Investig..

[bib109] Thambisetty M., Beason-Held L.L., An Y., Kraut M., Metter J., Egan J., Ferrucci L., O'Brien R., Resnick S.M. (2013). Impaired glucose tolerance in midlife and longitudinal changes in brain function during aging. Neurobiol. Aging.

[bib110] Tiedemann L.J., Schmid S.M., Hettel J., Giesen K., Francke P., Buchel C., Brassen S. (2017). Central insulin modulates food valuation via mesolimbic pathways. Nat. Commun..

[bib111] Tramutola A., Triplett J.C., Di Domenico F., Niedowicz D.M., Murphy M.P., Coccia R., Perluigi M., Butterfield D.A. (2015). Alteration of mTOR signaling occurs early in the progression of Alzheimer disease (AD): analysis of brain from subjects with pre-clinical AD, amnestic mild cognitive impairment and late-stage AD. J. Neurochem..

[bib112] Tschritter O., Preissl H., Hennige A.M., Stumvoll M., Porubska K., Frost R., Marx H., Klosel B., Lutzenberger W., Birbaumer N., Haring H.U., Fritsche A. (2006). The cerebrocortical response to hyperinsulinemia is reduced in overweight humans: a magnetoencephalographic study. Proc. Natl. Acad. Sci. USA.

[bib113] Unger J., Betz M. (1998). Invited Review-Insulin receptors and signal transduction proteins in the hypothalamo-hypophyseal system: A review on morphological findings and functional implications. Histol. Histopathol..

[bib114] Unger J., McNeill T.H., Moxley R.T., 3rd, White M., Moss A., Livingston J.N. (1989). Distribution of insulin receptor-like immunoreactivity in the rat forebrain. Neuroscience.

[bib115] Valla J., Jason D.B., Gonzalez-Lima F. (2001). Energy Hypometabolism in Posterior Cingulate Cortex of Alzheimer's Patients: Superficial Laminar Cytochrome Oxidase Associated with Disease Duration. J. Neurosci..

[bib116] Van Vugt D.A., Krzemien A., Alsaadi H., Frank T.C., Reid R.L. (2014). Glucose-induced inhibition of the appetitive brain response to visual food cues in polycystic ovary syndrome patients. Brain Res.

[bib117] Vanlandewijck M., He L., Mäe M.A., Andrae J., Ando K., Del Gaudio F., Nahar K., Lebouvier T., Laviña B., Gouveia L. (2018). A molecular atlas of cell types and zonation in the brain vasculature. Nature.

[bib118] Versteeg R.I., Koopman K.E., Booij J., Ackermans M.T., Unmehopa U.A., Fliers E., la Fleur S.E., Serlie M.J. (2017). Serotonin Transporter Binding in the Diencephalon Is Reduced in Insulin-Resistant Obese Humans. Neuroendocrinology.

[bib119] Wagner L., Veit R., Fritsche L., Haring H.U., Fritsche A., Birkenfeld A.L., Heni M., Preissl H., Kullmann S. (2022). Sex differences in central insulin action: Effect of intranasal insulin on neural food cue reactivity in adults with normal weight and overweight. Int J. Obes. (Lond. ).

[bib120] Wallum B.J., Taborsky G.J., Porte D., Figlewicz D.P., Jacobson L., Beard J.C., Ward W.K., Dorsa D. (1987). Cerebrospinal Fluid Insulin Levels Increase During Intravenous Insulin Infusions in Man. J. Clin. Endocrinol. Metab..

[bib121] Wang C., Reid G., Mackay C.E., Hayes G., Bulte D.P., Suri S. (2023). A systematic review of the association between dementia risk factors and cerebrovascular reactivity. Neurosci. Biobehav. Rev..

[bib122] Wang Z., Tang X., Swaminathan S.K., Kandimalla K.K., Kalari K.R. (2022). Mapping the dynamics of insulin-responsive pathways in the blood-brain barrier endothelium using time-series transcriptomics data. NPJ Syst. Biol. Appl..

[bib123] Wang Z.L., Zou L., Lu Z.W., Xie X.Q., Jia Z.Z., Pan C.J., Zhang G.X., Ge X.M. (2017). Abnormal spontaneous brain activity in type 2 diabetic retinopathy revealed by amplitude of low-frequency fluctuations: a resting-state fMRI study. Clin. Radio..

[bib124] Werther G.A., Hogg A., Oldfield B.J., McKinley M.J., Figdor R., Allen A.M., Mendelsohn F.A. (1987). Localization and characterization of insulin receptors in rat brain and pituitary gland using in vitro autoradiography and computerized densitometry. Endocrinology.

[bib125] Wever M.C.M., van Meer F., Charbonnier L., Crabtree D.R., Buosi W., Giannopoulou A., Androutsos O., Johnstone A.M., Manios Y., Meek C.L., Holst J.J., Smeets P.A.M., Full4Health c (2021). Associations between ghrelin and leptin and neural food cue reactivity in a fasted and sated state. Neuroimage.

[bib126] Wijtenburg S.A., Kapogiannis D., Korenic S.A., Mullins R.J., Tran J., Gaston F.E., Chen S., Mustapic M., Hong L.E., Rowland L.M. (2019). Brain insulin resistance and altered brain glucose are related to memory impairments in schizophrenia. Schizophr. Res..

[bib127] Willette A.A., Modanlo N., Kapogiannis D., Alzheimer's Disease Neuroimaging I. (2015). Insulin resistance predicts medial temporal hypermetabolism in mild cognitive impairment conversion to Alzheimer disease. Diabetes.

[bib128] Williams V.J., Trombetta B.A., Jafri R.Z., Koenig A.M., Wennick C.D., Carlyle B.C., Ekhlaspour L., Ahima R.S., Russell S.J., Salat D.H. (2019). Task-related fMRI BOLD response to hyperinsulinemia in healthy older adults. JCI Insight.

[bib129] Wingrove J., O'Daly O., De Lara Rubio A., Hill S., Swedroska M., Forbes B., Amiel S., Zelaya F. (2022). The influence of insulin on anticipation and consummatory reward to food intake: A functional imaging study on healthy normal weight and overweight subjects employing intranasal insulin delivery. Hum. Brain Mapp..

[bib130] Wingrove J., Swedrowska M., Scherlies R., Parry M., Ramjeeawon M., Taylor D., Gauthier G., Brown L., Amiel S., Zelaya F., Forbes B. (2019). Characterisation of nasal devices for delivery of insulin to the brain and evaluation in humans using functional magnetic resonance imaging. J. Control. Release.

[bib131] Wingrove J.O., O'Daly O., Forbes B., Swedrowska M., Amiel S.A., Zelaya F.O. (2021). Intranasal insulin administration decreases cerebral blood flow in cortico-limbic regions: A neuropharmacological imaging study in normal and overweight males. Diabetes, Obes. Metab..

[bib132] Wolf T., Tsenkova V., Ryff C.D., Davidson R.J., Willette A.A. (2018). Neural, Hormonal, and Cognitive Correlates of Metabolic Dysfunction and Emotional Reactivity. Psychosom. Med..

[bib133] Xia W., Wang S., Spaeth A.M., Rao H., Wang P., Yang Y., Huang R., Cai R., Sun H. (2015). Insulin Resistance-Associated Interhemispheric Functional Connectivity Alterations in T2DM: A Resting-State fMRI Study. Biomed. Res Int.

[bib134] Yang A.C., Vest R.T., Kern F., Lee D.P., Agam M., Maat C.A., Losada P.M., Chen M.B., Schaum N., Khoury N. (2022). A human brain vascular atlas reveals diverse mediators of Alzheimer’s risk. Nature.

[bib135] Yang X., Xu Y., Gao W., Wang L., Zhao X., Liu G., Fan K., Liu S., Hao H., Qu S., Dong R., Ma X., Ma J. (2022). Hyperinsulinemia-induced microglial mitochondrial dynamic and metabolic alterations lead to neuroinflammation in vivo and in vitro [Original Research]. Front. Neurosci..

[bib136] Young W.S. (1986). Periventricular hypothalamic cells in the rat brain contain insulin mRNA. Neuropeptides.

[bib137] Zhang H., Hao Y., Manor B., Novak P., Milberg W., Zhang J., Fang J., Novak V. (2015). Intranasal insulin enhanced resting-state functional connectivity of hippocampal regions in type 2 diabetes. Diabetes.

[bib138] Zhang Q., Zhang P., Yan R., Xu X., Mao C., Liu X., Li F., Ma J., Ye L., Yao Z., Wu J. (2019). A Single-Blinded Trial Using Resting-State Functional Magnetic Resonance Imaging of Brain Activity in Patients with Type 2 Diabetes and Painful Neuropathy. Diabetes Ther..

[bib139] Zhang W., Liu Q.Y., Haqqani A.S., Leclerc S., Liu Z., Fauteux F., Baumann E., Delaney C.E., Ly D., Star A.T. (2020). Differential expression of receptors mediating receptor-mediated transcytosis (RMT) in brain microvessels, brain parenchyma and peripheral tissues of the mouse and the human. Fluids Barriers CNS.

