# Supplementary material for: A systematic review of in vivo brain insulin resistance biomarkers in humans
Source: Biomark Neuropsychiatry. 2025 Jun;12:None. doi: 10.1016/j.bionps.2025.100125 (PMC13328063; doi:10.1016/j.bionps.2025.100125)
Supplement: Supplementary file 1 — Supplementary material [file mmc1.docx]

**1 ((brain or cerebral or neur* or central) adj5 insulin adj5 (resistance or signaling or action or receptor or binding or sensitiv*)).tw.**

**2 ((Intranasal adj5 insulin) or "HOMA*" or (homeosta* adj2 model adj2 assessment) or (hyperinsulin* adj4 clamp*) or "HbA1c" or "quantitative insulin sensitivity check index" or "QUIKI" or "HbA1c" or "glucose tolerance test" or "OGTT" or "insulin tolerance test" or "insulin sensitivity test").tw.**

**3 1 or 2**

**4 ("functional magnetic resonance imaging" or "fMRI" or "blood oxygen level dependent" or "BOLD" or "blood adj1 flow" or "resting adj1 state" or "arterial spin labelling" or "ASL" or "positron emission tomography" or "PET" or "electroencephalograph*" or "EEG" or "magnetoencephalograph*" or "MEG" or "functional near infrared spectroscopy" or "fNIRS" or "single photon emission comput* tomography" or "SPECT" or "doppler ultrasound" or "cranial ultrasound" or "transcranial doppler" or "single cell" or "electron microscopy" or "diffuse optical imaging" or "diffuse optical tomography" or "magnetic resonance spectroscop*" or "exosome" or "vesicl*").tw.**

**5 3 and 4**

**6 5 not (Animals/ not (Animals/ and Humans/))**
