# Supplementary material for: A systematic review of in vivo brain insulin resistance biomarkers in humans
Source: Biomark Neuropsychiatry. 2025 Jun;12:None. doi: 10.1016/j.bionps.2025.100125 (PMC13328063; doi:10.1016/j.bionps.2025.100125)
Supplement: Supplementary file 3 — Supplementary material [file mmc3.docx]

PRISMA Flow Chart Showing our Screening and Selection Process

**Identification of studies via other methods**

**Identification of studies via databases and registers**

Records removed *before screening*:

Duplicates removed (n = 2087)

6949 records identified from:

Medline (n = 1314)

EMBASE (n = 2910)

PsycINFO (n = 130)

CINAHL (n = 806)

Cochrane (n = 1494)

ProQuest (n = 295)

10 records identified from:

Citation searching (n = 10)

**Identification**

4618 records excluded:

Duplicates (n = 126)

No brain measure (n = 3062)

No IR measure (n = 563)

No association (n = 259)

Animal study (n = 404)

Review (n = 149)

Unpublished literature (n = 55)

Records screened

(n = 4862)

Reports not retrieved

(n = 0)

Reports sought for retrieval

(n = 10)

Reports sought for retrieval

(n = 244)

42 reports not retrieved:

No full text available (n = 22)

Unpublished literature (n = 20)

**Screening**

Reports assessed for eligibility

(n = 10)

6 Reports excluded:

No IR Comparison (n = 2)

No association (n = 3)

Review (n = 1)

Reports assessed for eligibility

(n = 202)

124 Reports excluded:

No brain measure (n = 11)

No IR measure (n = 45)

No association ( n = 8)

Unpublished literature (n = 60)

Studies included in review

(n = 82)

**Included**
