# Supplementary material for: A systematic review of in vivo brain insulin resistance biomarkers in humans
Source: Biomark Neuropsychiatry. 2025 Jun;12:None. doi: 10.1016/j.bionps.2025.100125 (PMC13328063; doi:10.1016/j.bionps.2025.100125)
Supplement: Supplementary file 5 — Supplementary material [file mmc5.docx]

| **Peripheral Insulin Resistance Measures** | | | | | | | | | | | |
| --- | --- | --- | --- | --- | --- | --- | --- | --- | --- | --- | --- |
| **Paper** | **ISO** | **Design** | **N** | **Subsamples** | **Age** | **BMI** | **Sex** | **Brain Insulin Resistance** | | **Findings** |  |
|  |  |  |  |  |  |  |  | **IR** | **Neuroimaging** |  |  |
| Akin et al. (2017) | TUR | ﻿  CS (BG) | 115 | ﻿  IR Obese: 31 IS Obese: 42  HC: 42 | 12.4 (2.3) 11.4 (2.4)  11.5 (2.3) | 2.1 (0.37)*  2.0 (0.29)*  0.26 (0.4)* | F = 17; M = 14 F = 18; M = 24  F = 21; M = 21 | HOMA-IR  6.1 (2.0)  2.6 (0.8)  1.5 (0.6) | EEG (frequency, % abnormality, slowing during hyperventilation, response to photic stimulation) | No differences across groups |  |
| Alsaadi and Van Vugt (2015) | CAN | CS (BG) | 19 | IR PCOS: 11  IS PCOS: 8 | 27.9 (5.84)  27.3 (4.65) | 40.0 (8.78)  30.8 (7.03) | F = 11; M = 0  F = 8; M = 0 | HOMA2-IR  2.3 (0.44)  0.9 (0.25) | BOLD fMRI | Positive association during glucose challenge to high calorie food in the right anterior cingulate cortex and left ventral tegmental area  Positive association during glucose challenge to low calorie food in the bilateral medial prefrontal cortex and right caudate  Positive association during glucose challenge to high calorie compared to low calorie foods in the bilateral midbrain |  |
|  |  |  |  |  |  |  |  |  |  | Negative association during glucose challenge to high calorie compared to low calorie foods in the bilateral dorsolateral prefrontal cortex |  |
| Anthony et al. (2006) | GBR | RM (BG) | 14 | IR: 7  IS: 7 | 49 (9.6) | 28.4 (23.7–35.3)  26.9 (23.9–30) | F = 0; M = 7  F = 0; M = 7 | HOMA-IR  6.3 (2.94–9.5)  1.3 (0.92–1.58) | FDG-PET | Smaller cerebral metabolic rate of glucose in the whole brain, right ventral striatum, and prefrontal cortex |  |
|  |  |  |  |  |  |  |  |  |  | No differences in cerebellum, amygdala, or left ventral striatum |  |
| Baker et al. (2011) | USA | CS (BG) | 29 | PD/T2DM: 23  HC: 6 | 74.4 (7.1)  74.3 (6.3) | 27.0 (2.9)  28.5 (3.4) | N/A  N/A | HOMA-IR  4.5 (N/A)  2.4 (N/A) | FDG-PET | Negative correlation between IR and cerebral metabolic rate of glucose in the posterior and medial cingulate, praecuneus, right parietal, left temporal, and left and right frontal lobes in patients |  |
|  |  |  |  |  |  |  |  |  |  | No correlation in healthy controls |  |
| Boersma et al. (2018) | SWE | CS (BG) | 35 | T2DM: 13  PD: 12  HC: 10 | 62 (7.0)  63 (7.0)  60 (6.0) | 30.4 (3.9)  31.0 (3.8)  30.7 (4.1) | F = 6; M = 7  F = 8; M = 4  F = 5; M = 5 | HEC (M-Value)  5.33 (0.71)  7.96 (1.17)  10.95 (1.23) | FDG-PET | Positive association between IR and whole-brain glucose uptake. Positive association between IR and glucose uptake in regions of interest, including secondary visual cortex, associative visual cortex, somatosensory association cortex, angular gyrus, fusiform gyrus, premotor cortex, and the inferior prefrontal gyrus |  |
| Castellano et al. (2015) | CAN | CS (BG) | 18 | PCOS: 7  HC: 11 | 24.6 (5.9)  24.0 (3.3) | 24.5 (2.4)  23.6 (3.0) | F = 7; M = 0  F = 11; M = 0 | HOMA2-IR  0.7 (0.3)  0.5 (0.3) | FDG-PET | Negative correlation between IR and cerebral metabolic rate of glucose in the superior frontal, superior parietal, and middle temporal lobes, as well as the hippocampus and amygdala across combined groups |  |
|  |  |  |  |  |  |  |  |  |  | No association between middle frontal, frontal pole, inferior parietal, supramarginal, superior temporal, inferior temporal, parahippocampal, entorhinal, thalamus, or caudate across combined groups |  |
| Castellano et al. (2019) | CAN | LON  (2 and 4 years) | 25 | Baseline: 25  2-year: 25  4-year: 16 | 70.9 (3.0)  73.1(3.0)  74.9 (2.4) | 27.7 (3.0)  27.0 (3.0)  27.3 (2.4) | F = 15; M = 10  F = 15; M = 10  F = 8; M = 8 | HOMA-IR  1.1 (0.5)  0.9 (0.5)  0.7 (0.4) | FDG-PET  C-AcAc PET | Negative correlation between IR and rate constant for brain extraction of glucose in the thalamus and caudate |  |
|  |  |  |  |  |  |  |  |  |  | No association between IR and rate constant for brain extraction of acetoacetate across any brain structures |  |
| Chen et al. (2022) | CHN | CS | 189 | IR = 34  IS = 34 | 48.68 (8.96)  N/A  N/A | 24.82 (2.92)  N/A  N/A | F = 60; M = 129  N/A  N/A | HOMA-IR  1.96 (1.59)  N/A  N/A | FDG-PET | Negative association between IR and whole-brain cerebral metabolic rate of glucose in the total sample  In a subsample of 34 matched controls, glucose metabolism was lower in IR compared to IS in bilateral middle temporal gyrus, bilateral middle frontal gyrus, right precentral gyrus, right inferior frontal gyrus, right cuneiform lobe and bilateral cerebellar  In the total sample, IR was negatively associated with glucose metabolism in right middle temporal gyrus, right cuneiform lobe, left middle temporal gyrus, and left middle frontal gyrus |  |
|  |  |  |  |  |  |  |  |  |  | Compared to matched controls, IR was associated with higher glucose metabolism in the right thalamus |  |
| Chen et al. (2014) | CHN | CS (BG) | 61 | T2DM: 30  HC: 31 | 59.0 (7.9)  57.2 (6.9) | 24.3 (2.8)  24.3 (2.3) | F = 14; M = 16  F = 15; M = 16 | HOMA-IR  3.54 (1.21)  1.50 (0.46) | rs-fMRI | Decreased connectivity between the posterior cingulate cortex and: the right middle temporal gyrus, the left lingual gyrus, the left middle occipital gyrus, and the left precentral gyrus  Negative association between IR and connectivity between posterior cingulate cortex and right middle temporal gyrus |  |
|  |  |  |  |  |  |  |  |  |  | Increased functional connectivity from the posterior cingulate cortex to the left cerebellum posterior lobe, the right superior frontal gyrus, and the right middle frontal gyrus |  |
|  |  |  |  |  |  |  |  |  |  | No association between glycated haemoglobin and functional connectivity between posterior cingulate cortex and the right middle temporal gyrus |  |
| Cui et al. (2015) | CHN | CS (BG) | 84 | T2DM:42  HC: 42 | 60.4 (7.0)  58.2 (6.3) | 24.7 (2.9)  24.0 (2.8) | F = 19; M = 23  F = 28; M = 14 | HOMA-IR  3.4 (2.0)  2.5 (1.3) | rs-fMRI | Decreased connectivity in posterior default mode network, including the posterior cingulate cortex and precuneus.  Negative association between IR and connectivity in posterior cingulate cortex in T2DM |  |
|  |  |  |  |  |  |  |  |  |  | Increased connectivity in anterior default mode network, including bilateral superior frontal gyrus around the medial prefrontal cortex |  |
|  |  |  |  |  |  |  |  |  |  | No association between IR and connectivity in HC  No association between IR and connectivity in anterior default mode network (bilateral superior frontal gyrus) or precuneus of posterior default mode network in T2DM |  |
| Cui et al. (2017) | CHN | CS (BG) | 81 | T2DM: 40  HC: 41 | 60.5 (6.9)  57.9 (6.5) | 24.4 (2.7)  23.8 (2.6) | F = 19; M= 21  F = 28; M = 13 | HOMA-IR  3.3 (1.9)  2.4 (1.1) | rs-fMRI | Decreased CBF in PCC, precuneus and bilateral middle occipital gyrus  Negative association between IR and CBF in posterior cingulate cortex and precuneus in T2DM |  |
|  |  |  |  |  |  |  |  |  |  | Increased CBF in dorsal anterior cingulate cortex |  |
|  |  |  |  |  |  |  |  |  |  | No difference in whole-brain CBF  No association between IR and CBF in HCs |  |
| Dai et al. (2017) | CHN | CS (BG) | 42 | DR: 21  HC: 21 | 55.9 (9.7)  55.4 (59.0) | N/A  N/A | F = 11; M = 10  F = 11; M = 10 | HOMA-IR  N/A  N/A | rs-fMRI | Positive association between IR and degree distribution in the right middle occipital gyrus |  |
|  |  |  |  |  |  |  |  |  |  | Negative association between IR and degree distribution in right superior orbital frontal gyrus, right middle frontal gyrus, right rolandic operculum, right insula, left angular gyrus, and right angular gyrus |  |
|  |  |  |  |  |  |  |  |  |  | No association between IR and clustering coefficient, characteristic path length, and small worldness in any brain region |  |
| Drummen et al. (2019) | NLD | CS | 39 | Obese: 39 | 53 (11.3) | 32.3 (3.7) | F = 22; M = 17 | HOMA-IR  3.9 (1.9) | BOLD fMRI | Positive correlation between IR and activity in the left nucleus accumbens, bilateral insula, and right cingulate gyrus when viewing food cues |  |
| Dunn et al. (2012) | USA | CS (BG) | 20 | Obese:12  HCs: 8 | 40 (8.0)  40 (9.0) | 40 (5.0)  23 (2.0) | F = 12; M = 0  F = 8; M = 0 | OGTT  3.9 (2.4)  11.2 (4.1) | Fallypride PET | Negative association between IR and availability of type 2 midbrain dopaminergic neurons in the left ventral striatum |  |
| Eckstrand et al. (2017) | USA | CS | 30 | N/A  (T2DM Obese) | 48.1 (6.9) | 36.5 (4.0) | F = 18; M = 12 | HOMA-IR  7.2 (4.1) | BOLD fMRI | Positive association between IR and activity in the right precuneus, right thalamus, left supplementary motor area, and right precentral gyrus during faster go speeds in stop signal tasks |  |
|  |  |  |  |  |  |  |  |  |  | Negative association between IR and activity in right putamen of the striatum during faster go speeds in stop signal tasks |  |
| Eriksson et al. (2021) | SWE | CS (BG) | 41 | T2DM: 13  PREDM: 16  HC: 12 | 63.0 (59.0–66.0)  65.5 (60.8–68.0)  60.5 (56.0–64.0) | 30.1 (28.0–33.9)  30.9 (28.2–32.5)  28.6 (26.8–33.5) | F = 6; M = 7  F = 9; M = 7  F = 6; M = 6 | HEC (GIR)  14.8 (12.0–27.8)  29.4 (18.5–31.8)  35.3 (29.9–43.8) | FDG-PET-MRI | Lower glucose infusion rates (higher IR) were associated with higher cerebral metabolic rate of glucose  Higher glycated haemoglobin (higher IR) were associated with higher cerebral metabolic rate of glucose |  |
| Femminella et al. (2021) | GBR | CS | 131 | N/A | 71.8 (7) | 25.9 (4.3) | F = 50; M = 80 | HOMA2-IR  1.1 (0.9) | FDG-PET | Negative association between IR and cerebral metabolic rate of glucose in the hippocampus |  |
| Garcia-Casares et al. (2014) | ESP | CS( BG) | 50 | T2DM: 25  HC: 25 | 60.0 (4.6)  57.8 (5.4) | 28.6 (4.1)  26.0 (3.2) | F = 8; M = 17  F = 11; M = 14 | HOMA-IR  3.6 (2.93)  1.4 (1.2) | FDG-PET | Decreased cerebral glucose metabolism in the left prefrontal and premotor areas, and bilateral middle and inferior temporal gyri  Negative association between IR and cerebral glucose metabolism in the left middle temporal gyrus and left insula in T2DM patients |  |
|  |  |  |  |  |  |  |  |  |  | No association between HbA1c and glucose metabolism |  |
| Gonzales et al. (2010) | USA | CS (BG) | 32 | Obese: 12  Overweight: 11  HC: 9 | 48.5 (8.6)  52.0 (5.1)  51.8 (4.3) | 34.3 (3.5)  27.4 (1.4)  22.4 (2.2) | F = 6; M = 6  F = 5; M = 6  F = 7; M = 2 | QUICKI  0.29 (0.02)  0.31 (0.02)  0.34 (0.03) | BOLD fMRI | Negative association between IR and activity in right parietal cortex during working memory 2-back task |  |
| Honkala et al. (2018) | FIN | RM (BG) | 21 | SIT: 10  MICT: 11 | 50 (46–54)  48 (44–52) | 29.9 (27.9–32.0)  30.9 (29.0–32.8) | F = 4; M = 6  F = 6; M = 5 | HEC (M-Value)  21.6 (14.5–28.8)  15.8 (9.1–22.4) | FDG-PET | Negative association between whole-body IR and brain glucose uptake across both exercise groups at baseline |  |
| Hoscheidt et al. (2017) | USA | CS (BG) | 120 | MetSyn: 30  HC: 90 | 58.7 (4.5)  56.4 (5.1) | N/A  N/A | F = 21; M = 9  F = 70; M = 20 | HOMA-IR  5.4 (6.1)  2.0 (1.3) | pcASL | Negative association between IR and CBF in the left middle frontal gyrus, the right anterior cingulate, the right precentral gyrus, the right medial frontal gyrus, and the right temporal lobe across groups |  |
| Ishibashi et al. (2017) | JPN | CS | 59 | N/A | 75.7 (6.4) | 18.5 – 25.0 | F = 49; M = 10 | HOMA-IR  1.0 (0.5) | FDG-PET | No association between IR and cerebral glucose uptake in the precuneus or lateral parietotemporal regions |  |
| Johansson et al. (2018) | SWE | CS (BG) | 10 | T2DM: 5  HC: 5 | 61 (57–71)  65 (42 – 68) | 28 (25 – 36)  26 (24 – 29) | F = 3; M = 2  F = 2; M = 3 | HEC (M-Value)  7.17 (1.96–16.00)  11.84 (5.54–15.00) | FDG-PET | Positive association between IR and glucose uptake in combined group of T2DM patients and HCs |  |
| Kullmann et al. (2012) | DEU | CS (BG) | 23 | Obese: 12  HC: 11 | 24.7 (2.4)  23.5 (2.1) | 30.5 (1.8)  20.9 (1.1) | F = 6; M = 6  F = 6; M = 5 | OGTT-DISI  11.5 (7.0)  19.0 (7.1) | rs-fMRI | Positive association between connectivity in left orbitofrontal cortex and IR  Positive association between connectivity in right putamen and IR |  |
| Latva-Rasku et al. (2018) | USA | CS (BG) | 45 | p.P50T/AKT2: 20  HC: 25 | 61.9 (6.3)  63.9 (4.8) | 28.7 (3.4)  28.1 (3.4) | F = 0; M = 20  F = 0; M = 25 | HEC (M-Value)  17.6 (10.3)  29.2 (15.2) | FDG-PET | Positive association between IR and brain glucose uptake across carriers and non-carriers  Compared to non-carriers, carriers had higher brain glucose uptake in all lobes, cerebellum, limbic system and midbrain |  |
| Lopez-Vilaret et al. (2022) | ESP | CS (BG) | 144 | PREDM: 71  HC: 74 | 67.65 (4.2)  67.6 (5.3) | 28.35 (3.75)  26.15 (3.25) | F = 40; M = 31  F = 39; M = 34 | HOMA-IR  2.95 (1.35)  1.75 (1.05) | rs-fMRI | Negative association between IR and short-range functional connectivity density of the left medial orbitofrontal cortex of the default mode network in PRDM |  |
|  |  |  |  |  |  |  |  |  |  | No differences in short-range or long-range functional connectivity density in regions of default mode network  No association in HC |  |
| Marder et al. (2014) | USA | CS (BG) | 51 | T2DM: 22  HC: 29 | 56.0 (5.5)  52.7 (5.5) | 30.1 (4.8)  28.6 (4.8) | F = 11; M = 11  F = 10; M = 19 | HOMA-IR  5.4 (5.8)  1.6 (1.4) | BOLD fMRI | Positive association between IR and activity in right middle frontal gyrus, right precentral gyrus, right inferior frontal gyrus during recognition task in healthy controls |  |
| Mazza et al. (2022) | ITA | CS | 92 | N/A  (Bipolar Disorder) | 47.86 (11.71) | 26.60 (4.92) | F = 57; M = 35 | HOMA-IR  3.16 (3.02)  QUICKI  0.34 (0.38) | rs-fMRI | Negative association between IR and activity in precuneus/ posterior cingulate cortex |  |
|  |  |  |  |  |  |  |  |  |  | No association between IR and functional connectivity |  |
| Musen et al. (2012) | USA | CS (BG) | 21 | T2DM: 10  HC: 11 | 56 (2.2)  54 (1.8) | 30.9 (2.1)  26.8 (1.3) | F = 3; M = 7  F = 4; M = 7 | HOMA-IR  7.4 (2.2)  2.6 (0.5) | rs-fMRI | Decrease connectivity between posterior cingulate cortex of default mode network and bilateral middle temporal gyrus, left medial and right inferior frontal gyri, and left thalamus  Negative association between IR and connectivity between posterior cingulate cortex and other default mode network regions, including right inferior frontal gyrus and right precuneus in T2DM |  |
| Nam et al. (2017) | KOR | CS (BG) | 264 | Low BGU: 67  Normal BGU: 197 | 47.6 (4.9)  45.6 (4.6) | 24.3 (2.8)  24.7 (3.0) | F = 0; M = 67  F = 0; M = 197 | HOMA-IR  1.37 (1.01)  1.15 (0.77) | FDG-PET | No difference in IR between people with low brain glucose uptake and normal levels of brain glucose uptake |  |
| Poessel et al. (2022) | DEU | CS (BG) | 53 | Obese: 19  Overweight: 10  HC: 24 | 29.7 (5.2)  30.1 (2.2)  27.5 (4.2) | 35.8 (4.7)  26.7 (1.3)  22.1 (1.6) | F = 9; M = 10  F = 5; M = 5  F = 12; M = 12 | HOMA-IR  1.86 (1.24)  0.80 (0.39)  0.67 (0.41) | BOLD fMRI | No association with activity in whole-brain, amygdala, orbitofrontal cortex, nucleus accumbens, ventral pallidum, ventral tegmental area, anterior cingulate cortex, insula, hippocampus, medial prefrontal cortex, puta- men, caudate nucleus, or piriform cortex during a olfactory-recognition test |  |
| Rebelos et al. (2020) | FIN | CS (BG) | 67 | T2DM: 15  HC: 52 | 49.0 (7.0)  45.0 (10.0) | 34.0 (12.1)^a^  27.4 (17.7)^a^ | F = 11; M = 4  F = 43; M = 9 | HEC (M-Value)  19.3 (14.7)^a^  39.4 (30.0)^a^ | FDG-PET | Positive association between IR and brain glucose uptake across T2DM and HC controls |  |
| Rebelos et al. (2021) | FIN | CS (BG) | 194 | Females: 163  Males: 63 | 56.0 (14.0)  56.0 (11.0) | 30.0 (7.0)  29.0 (6.0) | N/A | HEC (M-Value)  49.1 (25.3)  40.2 (24.5) | FDG-PET | Positive association between IR and brain glucose uptake in all four brain lobes across sexes |  |
| Rodriguez-Flores et al. (2014) | MEX | CS (BG) | 170 | Obese: 85  HC: 85 | 37 (27 – 44)  36 (26 – 44) | 41.2 (38.5 – 45)  22.9 (21.3 – 24.7) | F = 68; M = 17  F = 68; M = 17 | HOMA-IR  4.4 (2.9 – 6)  1.6 (1.1 – 2.2) | Doppler Ultrasound | Negative association between IR and cerebrovascular reactivity averaged across right and left middle cerebral arteries in both groups |  |
| Ryan et al. (2018) | USA | CS | 18 | N/A | 50.1 (4.9) | 26.6 (3.6) | F = 11; M = 7 | IVGTT  5.1 (2.1) | rs-fMRI | Positive relationship between IR and connectivity between left caudate and bilateral putamen following a meal  Positive relationship between IR and connectivity between the dorsal anterior cingulate cortex the left superior/medial frontal gyrus following a meal |  |
|  |  |  |  |  |  |  |  |  |  | Negative relationship between IR and centrality of the dorsal anterior cingulate cortex following a meal  Negative association between IR and connectivity between the dorsal anterior cingulate cortex and bilateral middle temporal gyrus, posterior cingulate cortex and occipital cortex in the fasted state  Negative association between IR and connectivity between dorsal anterior cingulate cortex and right postcentral gyrus following a meal |  |
|  |  |  |  |  |  |  |  |  |  | No association between IR and bilateral caudate connectivity in the fasted state  No association between IR and eigenvector centrality in the fasted state |  |
| Ryan et al. (2012) | USA | CS | 90 | N/A | 40.4 (6.4) | 28.33 (5.1) | F = 44; M = 46 | HOMA-IR  1.94 (N/A) | rs-fMRI | Positive association between IR and connectivity between left ventral striatum with left anterior insula dorsal left anterior mid-cingulate cortex |  |
| Thambisetty et al. (2013) | USA | LON (Baseline, 8 years) | 64 | IR: 15  IS: 49 | 59.7 (12.8)  56.5 (10.6) | 27.3 (3.2)  25.5 (3.0) | F = 7; M = 8  F = 19; M = 30 | OGTT  140 – 199  < 140 | O-Water PET | Increased CBF 8 years later in the left inferior frontal, left middle temporal, and left precentral gyri and the left globus pallidus |  |
|  |  |  |  |  |  |  |  |  |  | Decreased CBF 8 years later in right orbitofrontal cortex, right superior frontal gyrus, left superior temporal gyrus, left postcentral gyrus, right inferior parietal lobule, and the brainstem |  |
| Tschritter et al. (2006) | DEU | CS (BG) | 25 | Obese: 15  HC: 10 | 33 (2.0)  26 (1.0) | 29.7 (0.7)  21.1 (0.8) | F = 8; M = 7  F = 6; M = 4 | HEC-ISI  0.06 (0.01)  0.13 (0.01) | MEG | Negative association between IR and theta activity |  |
|  |  |  |  |  |  |  |  |  |  | No association between IR and beta activity |  |
| Van Vugt et al. (2014) | CAN | CS (BG) | 19 | IR: 8  IS: 11  PCOS | 29.3 (5.80)  26.5 (4.72) | 41.2 (8.93)  32.5 (7.75) | F = 8; M = 0  F = 11; M = 0 | OGTT  1.1 (0.26)  3.7 (2.35) | BOLD fMRI | Positive association between IR and brain activity when viewing high calorie food cues in inferior parietal lobule, posterior cingulate cortex, anterior cingulate cortex, precentral gyrus, middle frontal gyrus, middle temporal gyrus, superior frontal gyrus, precuneus, medial frontal gyrus and left cerebrum.  Positive association between IR and brain activity when viewing low calorie food cues in the precuneus, posterior cingulate cortex, superior frontal gyrus, superior temporal gyrus, rolandic operculum |  |
|  |  |  |  |  |  |  |  |  |  | Negative association between IR and brain activity when viewing high calorie food cues in the putamen |  |
| Versteeg et al. (2017) | NLD | CS (BG) | 20 | IR Obese: 6  IS Obese: 6  HC: 8 | 28.0 (6.6)  34.7 (9.9)  30.9 (10.5) | 38.3 (5.1)  34.9 (3.8)  21.3 (1.3) | F = 5; M = 1  F = 5; M = 1  F = 8; M = 0 | HOMA-IR  6.0 (4.8)  0.9 (0.3)  0.7 (0.5) | SPECT | Lower diencephalon serotonin transporter binding in IRO compared to ISO |  |
|  |  |  |  |  |  |  |  |  |  | No difference in hypothalamic serotonin transporter binding  No difference in dopaminergic transporter binding in the striatum |  |
| Wang et al. (2017) | CHN | CS (BG) | 37 | DR: 21  HC: 16 | 54.9 (9.9)  54.8 (5.7) | N/A  N/A | F = 11; M = 10  F = 9; M = 7 | HOMA-IR  3.37 (1.08)  2.34 (0.97) | rs-fMRI | Increased activity in inferior/middle/bilateral superior occipital gyrus, right lingual gyrus, and precuneus |  |
|  |  |  |  |  |  |  |  |  |  | Decreased activity in right posterior/anterior lobe of the cerebellum and the right fusiform, left para- hippocampal, right superior temporal, right inferior parietal, and angular gyrus |  |
|  |  |  |  |  |  |  |  |  |  | No association in DR patients between IR and activity in bilateral occipital gyrus, right lingual gyrus, precuneus, right posterior/anterior cerebellar lobe and the parahippocampal, fusiform, superior temporal, inferior parietal, and angular gyrus |  |
| Wever et al. (2021) | EU | CS | 109 | N/A | 49.56 (18.05) | 26.86 (4.70) | F = 56; M = 53 | HOMA-IR  2.54 (1.69) | BOLD fMRI | Positive association with activity in the left mid cingulate gyrus when viewing high calorie food in sated state  Positive association with activity in right rolandic operculum when viewing high calorie food in fasted state |  |
| Willette et al. (2015) | USA | LON (Baseline, 2 years) | 273 | AD: 60  Convert MCI: 39  Stable MCI: 148  HC: 26 | 75.69 (5.68)  75.83 (7.22)  75.26 (7.10)  75.69 (5.68) | 26.08 (3.83)  25.79 (3.56)  26.33 (3.91)  26.76 (3.48) | F = 38; M = 22  F = 25; M = 14  F = 102; M = 46  F = 15; M = 11 | HOMA-IR  0.60 (0.40)  0.26 (0.37)  0.30 (0.31)  0.87 (1.10) | FDG-PET | Positive association between IR and glucose metabolism in the medial temporal lobe in MCI patients who converted to AD |  |
|  |  |  |  |  |  |  |  |  |  | Negative association between IR and glucose metabolism in the medial temporal lobe in AD patients  Negative association between IR and glucose metabolism in the ventral prefrontal cortex in AD patients  Negative association between IR and glucose metabolism in the ventral prefrontal cortex in MCI who did convert to AD  Negative association between IR and glucose metabolism in the lateral and posteromedial parietal regions in AD patients |  |
|  |  |  |  |  |  |  |  |  |  | No association between IR and glucose metabolism in the medial temporal lobe for MCI or HC at baseline assessment  No association between IR and glucose metabolism in the medial temporal lobe for MCI who did not convert to AD  No association between IR and glucose metabolism in the ventral prefrontal cortex in MCI or HC at baseline assessment  No association between IR and glucose metabolism in the ventral prefrontal cortex in MCI converted to AD  No association between IR and glucose metabolism in the lateral and posteromedial parietal regions in MCI or HC at baseline  No association between IR and glucose metabolism in the lateral and posteromedial parietal regions regardless of MCI conversion |  |
| Williams et al. (2019) | USA | CS | 9 | N/A | 64.9 (7.7) | 25.0 (5.2) | F = 1; M = 8 | HEC-ISI  0.095 (0.041) | BOLD fMRI | Negative association between IR and activity in bilateral precuneus during n-back working memory task |  |
| Wolf et al. (2018) | USA | CS (BG) | 324 | T2DM: 65  PREDM: 109  HC: 150 | 58.35 (1.45)  53.33 (1.05)  50.55 (0.87) | 31.33 (0.88)  31.08 (0.60)  28.82 (0.54) | F = 38; M = 27  F = 54; M = 55  F = 89; M = 61 | HOMA-IR  0.74 (0.05)  0.52 (0.03)  0.30 (0.03) | EEG | Positive association between IR and right frontal asymmetry as measured by alpha wave frequency on f7/f8 output channels |  |
|  |  |  |  |  |  |  |  |  |  | No association between IR and right frontal asymmetry as measured by alpha wave frequency on f3/f4 output channels |  |
| Xia et al. (2014) | CHN | CS (BG) | 58 | T2DM: 30  HC: 28 | 59.5 (8.2)  56.2 (7.1) | 25.3 (2.7)  24.7 (2.4) | F = 14; M = 16  F = 15; M = 13 | HOMA-IR  3.8 (1.1)  1.5 (0.5) | rs-fMRI | Negative association between IR and interhemispheric connectivity of the middle temporal gyrus in T2DM |  |
|  |  |  |  |  |  |  |  |  |  | No association between IR and interhemispheric connectivity of the middle frontal gyrus, superior frontal gyrus, inferior parietal lobule, anterior cingulate gyrus, inferior occipital gyrus, or precentral gyrus |  |
| Zhang et al. (2019) | CHN | CS (BG) | 37 | PDN: 19  T2DM: 18 | 53.8 (8.1)  54.1 (6.9) | 24.84 (3.50)  24.46 (2.31) | F = 7; M = 12  F = 6; M = 12 | HOMA-IR  17.43 (10.44)  10.92 (6.64) | rs-fMRI | Negative association between IR and resting state activity in the left middle occipital lobe in patients with painful diabetic neuropath and left postcentral gyrus in T2DM without painful neuropathy |  |

* BMI is a sex and age specific BMI standard deviation score

^a^ Median and Interquartile Range

International Organisation for Standardisation Alpha 3 Country Codes

Abbreviations: Cross Sectional (CS), Between Groups (BG), Randomised Control Trial (RCT), Cross Over (CO), Within subjects (WS), Insulin Resistant (IR), Insulin Sensitive (IS), Progressive Supranuclear Palsy and Corticobasal Syndrome (PSP-CBS), Single-photon Emission Tomography (SPECT), Blood Oxygen Level Dependent (BOLD), Functional Magnetic Resonance Imaging (fMRI), Repeated Measures (RM), fluorodeoxyglucose-positron emission tomography (FDG-PET) Significant Stenosis (S-STN), Non-Significant Stenosis, Polycystic Ovary Syndrome (PCOS), C-acetoacetate positron emission tomography (C-AcAc PET), Type-2 Diabetes Mellitus (T2DM), Healthy Controls (HC), Pseudo-continuous Arterial Spin Labelling (pcASL), Cerebral Blood Flow (CBF), Resting State Functional Magnetic Resonance Imaging (rs-fMRI), dimethylaminomethylphenylsulfanyl-benzonitrile positron emission tomography (DASB-PET), Metabolic Syndrome (MetSyn), Sprint Interval Training (SIT), Moderate Intensity Continuous Training (MICT), Diabetic Retinopathy (DR), Magnetic Resonance Spectroscopy (MRS), Intravenous Glucose Tolerance Test (IVGTT), Hyperinsulinemic-Euglycemic Clamp – Insulin Sensitivity Index (HEC-ISI)
