# Supplementary material for: A systematic review of in vivo brain insulin resistance biomarkers in humans
Source: Biomark Neuropsychiatry. 2025 Jun;12:None. doi: 10.1016/j.bionps.2025.100125 (PMC13328063; doi:10.1016/j.bionps.2025.100125)
Supplement: Supplementary file 6 — Supplementary material [file mmc6.docx]

| **Intranasal Responsiveness Measures** | | | | | | | | | | | |
| --- | --- | --- | --- | --- | --- | --- | --- | --- | --- | --- | --- |
| **Paper** | **ISO** | **Design** | **N** | **Subsamples** | **Age** | **BMI** | **IR** | **Sex** | **Brain Insulin Resistance** | | **Findings** |
|  |  |  |  |  |  |  |  |  | **Dose** | **Neuroimaging** |  |
| Akintola et al. (2017) | NLD | RCT (CO) | 19 | Older: 11  Younger: 8 | 65.2 (3.3)  22.3 (1.8) | 24.1(2.2)  23.6(2.2) | HOMA-IR  1.05 (0.65 -2.03)*  1.21 (1.00-1.49)* | F = 0; M = 11  F = 0; M = 8 | 40 IU/ 0.4 mL (Saline Placebo) | pcASL | Increase in occipital and thalamic CBF in older adults |
|  |  |  |  |  |  |  |  |  |  |  | No change in other lobes or the hypothalamus in older or younger adults |
| Brunner et al. (2016) | DEU | RCT  (CO) | 11 | N/A | 24.91 (1.30) | 23.69 (0.22) | N/A | F = 0; M =11 | 40 IU/ 0.4 mL (Unspecified Placebo) | BOLD fMRI | No change in activity during olfactory or visual maze tasks |
| Craft et al. (2012) | USA | LON RCT  (2 and 4 months) | 104 | Placebo: 30  20 IU: 36  40 IU: 38  AD: 40  MCI: 64 | 74.9 (1.6)  72.8 (1.5)  69.9 (1.4) | 27.4 (0.8)  26.7 (0.8)  26.9 (0.7) | N/A | F= 13; M = 17  F = 14; M = 22  F = 18; M = 20 | Two half doses per day  (Unspecified placebo) | FDG-PET | Both 20 IU and 40 IU associated with reduced progression of hypometabolism in bilateral frontal, right temporal, bilateral occipital, and right precuneus and/or cuneus  20 IU associated with reduced progression of hypometabolism in right temporal lobe  40 IU associated with reduced progression of hypometabolism in the left parietal cortex |
|  |  |  |  |  |  |  |  |  |  |  | No association between 40 IU and hypometabolism in temporal cortex |
| Grichisch et al. (2012) | DEU | RCT (CO) | 8 | N/A | 18-34 | 20-25 | N/A | F = 5; M = 3 | 160 IU/ 0.4 mL  (200mg caffeine control) | Pulsed ASL | No change in CBF between baseline and post-insulin administration during task-free and flickering checkerboard conditions in the visual cortex, right anterior, or left posterior parts of the brain |
| Guthoff et al. (2010) | DEU | RCT (CO) | 9 | N/A | 24.6 (1.3) | 21.4 (0.7) | HbA1c  5.2 (0.1) | F = 4; M = 5 | 160 IU/ 0.4 mL  (Unspecified Placebo) | BOLD fMRI | Reduced activity compared to baseline in right and left fusiform gyrus, the right hippocampus, the right temporal superior cortex, and the right frontal middle cortex when viewing food pictures |
|  |  |  |  |  |  |  |  |  |  |  | No effect of placebo on activity when viewing food and non-food stimuli |
| Guthoff et al. (2011) | DEU | RCT (CO) | 20 | Obese: 10  HC: 10 | 26.7 (1.8)  25.7 (1.5) | 28.8 (0.6)  20.9 (0.4) | HbA1c  5.5 (0.1)  5.4 (0.1) | F = 7; M = 3  F = 7; M = 3 | 160 IU/ 1.6 mL  HOE 31 Dilution Buffer | MEG | Increased M2 (ventral stream, focusing on FFA) component for food pictures in lean subjects |
|  |  |  |  |  |  |  |  |  |  |  | No effect of insulin on M1 (primary visual cortex) for food or non-food pictures in lean or obese subjects. No effect of insulin on M2 for non-food pictures in lean or obese subjects |
| Heni et al. (2016) | DEU | CS (WS) | 45 | N/A | 26.4 (3.4) | N/A | N/A | F = 19; M = 26 | 160 IU/ 0.4 mL  No placebo | rs-fMRI | Decreased activity in caudate nucleus of striatum in FTO non-risk allele carriers with ANK11 D2 receptor risk allele |
|  |  |  |  |  |  |  |  |  |  |  | Increased activity in caudate nucleus of striatum in FTO obesity-risk allele carriers with ANK11 D2 receptor risk allele |
|  |  |  |  |  |  |  |  |  |  |  | No difference in activity between FTO polymorphisms in participants with ANK11 D2 receptor non-risk allele, but caudate activity decreased in both groups |
| Ketterer et al. (2014) | DEU | RCT (CO) | 43 | N/A | 33.0 (10) | 22.9 (3.2) | HOMA-IR  1.4 (N/A) | F = 20; M = 23 | 160 IU/ 1.6 mL  HOE 31 Dilution Buffer | MEG | Increased theta band power during food-related working memory task in carriers of rs3123554 single nucleotide polymorphism in cannabinoid-receptor 2 gene (CNR2)  Smaller increase in homozygous carriers of minor allele (AA) compared to carriers of major allele (GG or AG) |
| Kleinloog et al. (2022) | NLD | CS (WS) | 18 | N/A | 50 (23 – 60) | 33.4 (5.0) | N/A | F = 0; M = 18 | 160 IU/ 1.6 mL  No Placebo | pcASL | Increased subcortical CBF compared to baseline |
|  |  |  |  |  |  |  |  |  |  |  | No difference in whole-brain, grey matter or cortical CBF compared to baseline |
| Kullmann et al. (2021) | DEU | RCT (CO) | 10 | N/A | 27 (3) | 23.6 (2.3) | HOMA-IR  1.8 (1.1) | F = 0; M = 10 | 160 IU/ Unspecified Volume  Vehicle Placebo | CR-PET  rs-fMRI | Increased binding potential to D2 and D3 receptors in ventral and dorsal striatum (caudate and putamen) |
|  |  |  |  |  |  |  |  |  |  |  | Decreased resting state activity in caudate |
| Kullmann et al. (2013) | DEU | RCT (CO) | 17 | N/A | 24.47 (2.21) | 21.16 (1.64) | N/A | F = 17; M = 0 | 160 IU/ 0.4 mL  Vehicle Placebo | rs-fMRI | Decreased activity in left hypothalamus and left orbitofrontal cortex |
| Kullmann et al. (2015) | DEU | RCT (CO) | 48 | Obese: 23  HC: 25 | 26.73 (3.55)  25.88 (3.30) | 31.26 (4.77)  22.65 (2.01) | OGTT-DISI  10.24 (6.72)  16.00 (7.60) | F =11 ; M = 12  F = 10; M = 15 | 160 IU/ Unspecified Volume  Vehicle Placebo | pcASL | Decreased CBF in right middle frontal gyrus in HC  Decreased CBF in hypothalamus in obese and HC |
|  |  |  |  |  |  |  |  |  |  |  | No change in CBF in right middle frontal gyrus in obese patients |
| Kullmann et al. (2017) | DEU | RCT (CO) | 47 | Obese: 22  HC: 25 | 26.81 (3.62)  25.88 (3.30) | 30.57 (3.51)  22.59 (1.99) | OGTT-DISI  10.66 (6.56)  16.0 (7.6) | F =11 ; M = 11  F = 10; M = 15 | 160 IU/ Unspecified Volume  Vehicle Placebo | rs-fMRI | Increased connectivity between the anterior medial prefrontal cortex and the right hippocampus and between the dorsal medial prefrontal cortex and the right hippocampus in both groups  Increased connectivity between anterior medial prefrontal cortex and the hypothalamus in participants with high peripheral insulin sensitivity |
|  |  |  |  |  |  |  |  |  |  |  | No change in connectivity between anterior medial prefrontal cortex and the hypothalamus in participants with low peripheral insulin sensitivity |
| Novak et al. (2014) | USA | RCT (CO) | 29 | T2DM: 15  HC: 14 | 62.0 (7.9)  60.1 (9.9) | N/A  N/A | N/A  N/A | F = 7; M = 8  F = 10; M = 4 | 40 IU/ 0.4 mL  Saline Placebo | pcASL | Increased CBF in right insular cortex in T2DM  Greater CBF increase in right insular cortex in T2DM compared to HC |
| Novak et al. (2022) | USA | LON RCT  (BS) | 11 | N/A  T2DM | N/A | N/A | N/A | N/A | 40 IU/ 0.4 mL/ day  0.9% BSC/ 0.4 mL/ day | pcASL | Increased CBF after 24 weeks in right medio-prefrontal cortex |
|  |  |  |  |  |  |  |  |  |  |  | Smaller hypercapnia-induced increases in CBF after 24 weeks in the posterior cingulate cortex/precuneus  Smaller hypocapnia-induced decreases in CBF after 24 weeks in the occipito-parietal junction |
| Opstal et al. (2017) | NLD | RCT (CO) | 8 | N/A | 22.3 (1.8) | 23.6 (2.2) | 1.2 (1.0 -1.5)* | F = 0; M = 8 | 40 IU/ Unspecified Volume  Saline Placebo | BOLD fMRI | Decreased hypothalamic activity during glucose ingestion compared to placebo |
| Schilling et al. (2014) | DEU | RCT (BS) | 48 | N/A | 23.98 (3.4) | 20-25 | N/A | F = 0; M = 48 | 40 IU/ 0.4 mL  Dilution Buffer Placebo | pcASL | Increased CBF in bilateral insular cortex, bilateral putamen, left caudate nucleus, and opercular part of the bilateral inferior frontal gyrus |
|  |  |  |  |  |  |  |  |  |  |  | No effect on CBF in the calcarine fissure |
| Stingl et al. (2010) | DEU | RCT (CO) | 20 | Obese: 10  HC: 10 | 26.7 (1.8)  25.7 (1.5) | 28.8 (0.6)  20.9 (0.4) | HbA1c  5.5 (0.1)  5.4 (0.1) | F = 7; M =3  F = 7; M = 3 | 40 IU/ 0.4 mL  HOE 31 dilution buffer | MEG | Increased theta (4-8Hz) path length (global interconnectedness) in obese participants |
|  |  |  |  |  |  |  |  |  |  |  | Decreased theta (4-8Hz) path length (global interconnectedness) in HC |
| Edwin Thanarajah et al. (2019) | DEU | RCT (CO) | 36 | IR: 17  IS: 19 | 27.77 (4.51)  29.51 (4.58) | 25.09 (2.50)  25.23 (2.81) | HOMA-IR  2.44 (1.02)  1.07 (0.23) | F = 0; M = 17  F = 0; M = 19 | 40 IU/ 0.4 mL  100 IU/ 1.0 mL  160 IU/ 1.6 mL  Saline Placebo | rs-fMRI | Compared to placebo, 40 IU and 160 IU increased connectivity between ventral tegmental area and ventromedial prefrontal cortex 25-35 minutes after administration in low peripheral insulin resistance  Compared to placebo, 40 IU and 100 IU increased connectivity between ventral tegmental area and ventromedial prefrontal cortex 80-90 minutes after administration in high peripheral insulin resistance |
|  |  |  |  |  |  |  |  |  |  |  | Compared to placebo, 100 IU decreased connectivity between ventral tegmental area and ventromedial prefrontal cortex at baseline in high peripheral insulin resistance |
| Tiedemann et al. (2017) | DEU | RCT (CO) | 48 | IR: 20  IS: 28 | 26.1 (0.7)  25.7 (0.7) | 29.4 (1.1)  23.6 (0.7) | HOMA-IR  2.4 (0.2)  1.2 (0.1) | F = 11; M = 9  F = 14; M = 14 | 160 IU/ 1.6 mL  Water, 0.27% m-Kresol, 1.6% Glycerol Placebo | BOLD fMRI | Increased activity in nucleus accumbens and left ventral tegmental area when viewing food images in IR |
|  |  |  |  |  |  |  |  |  |  |  | Decreased activity in nucleus accumbens and left ventral tegmental area when viewing food images in IS |
| Wagner et al. (2022) | DEU | RCT (CO) | 60 | Obese F: 10  Obese M: 13  HC F: 20  HC M: 17 | 42.9 (5.35)  47.08 (3.9)  42.55 (3.41)  40.29 (3.99) | 27.5 (0.7)  27.01 (0.5)  22.9 (0.25)  22.66 (0.49) | OGTT  18.84 (4.78)  14.78 (2.37)  16.41 (1.6)  18.9 5 (3.13) | F = 10; M = 0  F = 0; M = 13  F = 20; M = 0  F = 0; M = 17 | 160 IU/ Unspecified Volume  Vehicle Placebo | BOLD fMRI | Increased amygdala activity in all groups when viewing high-calorie food  Increased precuneus and insular activity in healthy males and overweight females when viewing high-calorie food  Increased activity in the dorsolateral prefrontal cortex in females in response to highly desired food cues |
|  |  |  |  |  |  |  |  |  |  |  | Decreased precuneus and insular activity in healthy females and overweight males when viewing high-calorie food  Decreased activity in the dorsolateral prefrontal cortex in men in response to highly desired food cues |
| Wingrove et al. (2022) | GBR | RCT (CO) | 24 | Obese: 14  HC: 10 | 25.0 (4.3)  27.0 (5.4) | 27.5 (1.7)  22.4 (1.9) | HOMA-IR  1.04 (0.37)  0.97 (0.22) | F = 0; M = 14  F = 0; M = 10 | 160 IU/ 0.4 mL  Saline Placebo | BOLD fMRI | Increased activity in anterior cingulate cortex to sugar and stevia cues in the HC compared to the overweigh participants  Increased activity in the nucleus accumbens to stevia cues in the HC compared to overweight participants |
|  |  |  |  |  |  |  |  |  |  |  | Decreased activity in the amygdala when consuming sugar in the HC compared to the overweight participants |
| Wingrove et al. (2019) | GBR | RCT (CO) | 16 | N/A | 24.67 (4.30) | 27.76 (1.92) | NA/ | F = 0; M = 16 | 160 IU/ 0.4 mL  Saline Placebo | pcASL | Decreased CBF in bilateral amygdala |
|  |  |  |  |  |  |  |  |  |  |  | No effect on CBF in whole brain or bilateral hippocampus |
| Wingrove et al. (2021) | GBR | RCT (CO) | 26 | Obese: 14  HC: 12 | 24.76 (4.30)  27.00 (5.44) | 27.76 (1.92)  22.40 (1.89) | HOMA-IR  1.04 (0.37)  0.87 (0.22) | F = 0; M = 14  F = 0; M = 12 | 160 IU/ 0.4 mL  Saline Placebo | pcASL | Decreased CBF in bilateral parahippocampal gyrus, right fusiform gyrus, left insula/putamen, left hippocampus in overweight group |
|  |  |  |  |  |  |  |  |  |  |  | No effect on CBF in whole brain in either group |
| Zhang et al. (2015) | CHN | RCT (CO) | 28 | T2DM: 14  HC: 14 | 61.7 (8.1)  60.1 (9.9) | N/A | N/A | F = 6; M = 8  F = 10; M = 4 | 40 IU/ 0.4 mL  Saline Placebo | rs-fMRI | Increased functional connectivity between hippocampus and default mode network (medial frontal cortex, posterior cingulate cortex, and anterior cingulate cortex) in T2DM and HCs  Increase cluster size of medial frontal cortex connected to right hippocampus in T2DM  Increased correlation between right hippocampus and right inferior parietal cortex in T2DM  Increased cluster size of left posterior cingulate cortex connected to the left hippocampal in HC |
|  |  |  |  |  |  |  |  |  |  |  | No effect of insulin on cluster size of left medial frontal cortex connected to left hippocampus in T2DM  No effect of insulin on correlation between left anterior cingulate cortex and hippocampus in HC |

* Median (IQR)

Abbreviations: Pseudo Continuous Arterial Spin Labelling (pcASL), Blood Oxygen Level Dependent (BOLD), Functional Magnetic Resonance Imaging (fMRI), magnetoencephalography (MEG), Magnetic Resonance Spectroscopy (MRS), [11C]-raclopride Positron Emission Tomography (CR-PET), Oral Glucose Tolerance Test Derived Insulin Sensitivity Index (OGTT-DISI), Bacteriostatic Sodium Chloride (BSC)
