# Supplementary material for: A systematic review of in vivo brain insulin resistance biomarkers in humans
Source: Biomark Neuropsychiatry. 2025 Jun;12:None. doi: 10.1016/j.bionps.2025.100125 (PMC13328063; doi:10.1016/j.bionps.2025.100125)
Supplement: Supplementary file 7 — Supplementary material [file mmc7.docx]

| **Neuronally Enriched Extracellular Vesicles** | | | | | | | | | | | | | | |
| --- | --- | --- | --- | --- | --- | --- | --- | --- | --- | --- | --- | --- | --- | --- |
| **Paper** | **ISO** | **Design** | **N** | **Subsamples** | **Age** | **BMI** | **IR** | **Sex** | **Brain Insulin Signalling** | | | | | **Findings** |
|  |  |  |  |  |  |  |  |  | **Source** | **Isolation** | **Detection** | **Proteins** | **Measure** |  |
| Athauda et al. (2019) | GBR | LON RCT  (24, 48, and 60 weeks) | 60 | PD-Exenatide: 31  PD-Placebo: 29 | 61.5 (8.3)  58.3 (8.5) | N/A  N/A | N/A  N/A | F = 9; M = 21  F = 7; M = 22 | Serum | PPP + L1CAM-IC | N/A | pY-IRS-1, pSer616-IRS-1, pSer312-IRS-1, and total (t-) and phosphorylated (p-) forms of Akt, mTOR, GSK3β, p38-MAPK, ERK1/2, and JNK | ECI | Increased pY-IRS-1, pSer616-IRS-1, pSer312-IRS-1, t-Akt, p-Akt S473, and p-mTOR in response to exenatide |
|  |  |  |  |  |  |  |  |  |  |  |  |  |  | No change in t-mTOR, t-GSK3β, p-GSK3β, t-p38 or p-p38-MAPK, ERK1/2, or JNK in response to exenatide |
| Avgerinos et al. (2022) | USA | LON (Baseline, 14 days) | 21 | Empagliflozin: 21 | 62.14 (6.91) | 27.15 (2.97) | HOMA-IR  1.54 (1.30) | F = 11; M = 10 | Plasma | PPP + L1CAM-IC | cTEM | pIGF-1R, pIR, pIRS-1,  pSer312-IRS-1, p-JNK, p-p38-MAPK, pERK1/2, p-Akt, and t-Akt | ECI | Increased pIGF-1R, pIR, pY-IRS-1, p-Akt after a single dose of Empagliflozin  Increased p-p38-MAPK after 14 days of Empagliflozin |
|  |  |  |  |  |  |  |  |  |  |  |  |  |  | Decreased (pSer312-IRS-1)/  (pY-IRS-1), p-JNK after 14 days of Empagliflozin |
|  |  |  |  |  |  |  |  |  |  |  |  |  |  | No effect of Empagliflozin on pSer312-IRS-1, p-ERK1/2 or t-Akt at any point |
| Blommer et al. (2022) | NZL | CS (BG) | 273 | PD-Dementia: 40  PD-MCI: 81  PD-Normal: 103  HC: 49 | 76.2 (5.2)  72.0 (6.8)  69.9 (7.1)  75.8 (7.3) | 26.3 (4.4)  25.8 (4.2)  25.7 (4.3)  26.3 (4.0) | N/A  N/A  N/A  N/A | F = 7; M = 33  F = 23; M = 58  F = 38; M = 65  F = 21; M = 28 | Plasma | PPP + L1CAM-IC | cTEM, NTA | pSer312-IRS-1, pY-IRS-1, mTOR, and p-mTOR | ECI | Increased (pSer312-IRS-1)/ (pY-IRS-1) in PD compared to HC and PD-MCI compared to PD-Normal and HC |
|  |  |  |  |  |  |  |  |  |  |  |  |  |  | Decreased pY-IRS-1 in PD compared to HC, PD with any cognitive impairment compared to PD-Normal and HC, and PD-MCI compared to HC |
|  |  |  |  |  |  |  |  |  |  |  |  |  |  | No difference in pSer312-IRS-1 between groups  No difference in mTOR or p-mTOR between groups  No difference in pY-IRS-1 between PD-Normal or PD-dementia and HC, PD-MCI or PD-Dementia and PD-Normal, or PD-Dementia and PD-MCI  No difference in (pSer312-IRS-1)/ (pY-IRS-1) between PD-Normal and HC, PD-Dementia and PD-MCI or PD-Normal |
| Kapogiannis et al. (2015) | USA | CS (BG) | 124 | AD: 26  HC: 26  FTD: 16  HC: 16  T2DM: 20  HC: 20 | 74.3 (7.48)  74.3 (7.48)  63.1 (8.79)  63.7 (7.43)  73.0 (9.27)  73.0 (9.19) | N/A  N/A  N/A  N/A  N/A  N/A | N/A  N/A  N/A  N/A  N/A  N/A | F = 13; M = 13  F = 13; M = 13  F = 4; M = 12  F = 4; M = 12  F = 11; M = 9  F = 11; M = 9 | Plasma | PPP + L1CAM-IC | N/A | pSer312-IRS-1, pY-IRS-1, IRS-1 | ELIA | Increased pSer312-IRS-1 in AD and T2DM compared to HC  Increased IRS-1 in AD compared to HC  Increased (pSer312-IRS-1)/  (pY-IRS-1) in AD and T2DM compared to HC |
|  |  |  |  |  |  |  |  |  |  |  |  |  |  | Decreased pY-IRS-1 in AD and T2DM compared to HC |
|  |  |  |  |  |  |  |  |  |  |  |  |  |  | No difference in IRS-1 for FTD or T2DM compared to HCs |
| Kapogiannis, Dobrowolny, et al. (2019) | DEU | CS (BG) | 48 | SCZ: 24  HC: 24 | 32.75 (11.76)  35.00 (10.70) | 23.89 (4.11) 24.31 (3.33) | HOMA-IR  1.46 (1.78)  0.52 (0.42) | F = 11; M = 13  F = 9; M = 15 | Plasma | PPP + L1CAM-IC | N/A | pSer312-IRS-1, pY-IRS-1, p-AKT, p-GSK3β, p-mTOR, p-p70S6K  and total for each | ECI | Decreased p/t ratio in SCZ compared to HC  Decreased mTOR/ p-mTOR ratio in SCZ compared to controls |
|  |  |  |  |  |  |  |  |  |  |  |  |  |  | No difference in pSer312-IRS-1 or pY-IRS between SCZ and HC |
| Kapogiannis, Mustapic, et al. (2019) |  | LON (Baseline, 4 years) | 350 | Future AD: 128  HC: 222 | 79.09 (7.02)  76.20 (7.36) | N/A  N/A | N/A  N/A | F = 68; M = 60  F = 110; M = 111 | Plasma  Serum | PPP + L1CAM-IC | NTA, EM, WB | pSer312-IRS-1, pY-IRS-1 | ECI | Increased pSer312-IRS-1 and pY-IRS-1 in patients with future AD across all clinical visits compared to HC |
| Mansur et al. (2021) | CAN | LON RCT (Baseline, 2, 6, and 12 weeks) | 55 | BD-Infliximab: 27  BD-Placebo: 28 | 44.04 (11.55)  45.75 (10.28) | 34.57 (10.08)  34.55 (7.66) | N/A  N/A | F = 20; M = 7  F = 24; M = 4 | Plasma | PPP + L1CAM-IC | NTA, WB | pSer312-IRS-1, p-ERK1/2, p-JNK, p-p38-MAPK, p-Akt, p-GSK3β, p-p70S6K | ECI | Increase in factor scores for factor the alternative insulin signalling pathway (pSer312-IRS-1, p-ERK1/2, p-JNK and p-p38-MAPK) in response to infliximab  Increase in p-ERK1/2, p-JNK and p-p38-MAPK in response to infliximab compared to placebo  Increased p-JNK in infliximab responders compared to non-responders and placebo  Increased p-ERK1/2 in infliximab responders compared to placebo non-responders |
|  |  |  |  |  |  |  |  |  |  |  |  |  |  | No effect on factor scores for the factor representing the canonical insulin signalling pathway (p-Akt, p-GSK-3β, p-p70S6K) in response to infliximab |
| Mustapic et al. (2019) | USA | LON RCT (Baseline, 4 months) | 91 | Placebo: 26  20 IU INI: 33  40 IU INI: 32  AD/MCI Patients | 76.2 (9.06)  76.2 (9.06)  69.6 (9.13) | N/A  N/A  N/A | N/A  N/A  N/A | F = 15; M = 11  F = 21; M = 12  F = 20; M = 12 | Plasma | PPP + L1CAM-IC | NTA | pSer312-IRS-1, pSer616-IRS-1, t-IRS-1, pY-IRS-1, p-AKT, p-GSK3β, p-S6RP, p-p70S6K, t-mTOR, p-mTOR, p-ERK1/2, p-JNK, and p-p38-MAPK | ECI | Increased p-Akt and p-S6RP following 20 and 40 IU after 4 months |
|  |  |  |  |  |  |  |  |  |  |  |  |  |  | No effect on IRS-1 or any other downstream proteins involved in insulin signalling pathways |
| Singh et al. (2022) | DEU | CS (BG) | 42 | MD: 21  HC: 21 | 43.0 (12.8)  37.3 (13.0) | 24.0 (4.4)  23.6 (3.5) | HOMA-IR  0.68 (0.63)  0.90 (1.14) | F = 8; M = 13  F = 8; M = 13 | Plasma | L1CAM-IC | N/A | pSer312-IRS-1, pY-IRS-1, p-Akt, p- GSK3β, p-mTOR, p-p70S6K, Akt, GSK3β, mTOR, and p70S6K | ECI | No difference in pSer312-IRS-1/pY-IRS-1 ratio between MD and HC  No difference in downstream proteins between MD and HC |
| Wijtenburg et al. (2019) | USA | CS (BG) | 46 | SCZ: 22  HC: 24 | 39.5 (14.5)  36.7 (16.0) | N/A  N/A | N/A  N/A | F = 7; M = 15  F = 10; M = 14 | Plasma | PPP + L1CAM-IC | NTA | Akt, GSK3β, p70S6K, p-Akt, p-GSK3β, and p-p70S6K | ECI | No differences in any downstream proteins between SCZ and HC |

Abbreviations: Parkinson’s Disease (PD), Healthy Control (HC), Polymer Particle Precipitation (PPP), L1 Cell Adhesion Molecule Immunocapture (L1CAM-IC), Cryogenic Transmission Electron Microscopy (cTEM), Nanoparticle Tracking Analysis (NTA), Electron Microscopy (EM), Tyrosine Phosphorylated Insulin Receptor Substrate 1 (IRS-1 p-Tyr), Phosphorylation at Serine Positions (p-S), Protein Kinase B (Akt), Mechanistic Target of Rapamycin (mTOR), Glycogen Synthase Kinase 3β (GSK-3β), Mitogen-Activated Protein Kinase (MAPK), Extracellular Signal-Related Kinase (Erk), c-Jun N-terminal kinase (JNK), Insulin-like Growth Factor 1 Receptor (IGF-1R), Schizophrenia (SCZ), Major Depression (MD), Frontotemporal Dementia (FTD), Western Blot (WB), Electrochemiluminescence (ECI), Enzyme-Linked Immunosorbent Assay (ELIA)
